# *N-Acetyltransferase 9* ameliorates Aβ42-mediated neurodegeneration in the *Drosophila* eye

**DOI:** 10.1038/s41419-023-05973-z

**Published:** 2023-07-28

**Authors:** Prajakta Deshpande, Anuradha Venkatakrishnan Chimata, Emily Snider, Aditi Singh, Madhuri Kango-Singh, Amit Singh

**Affiliations:** 1 Department of Biology, University of Dayton, Dayton, OH 45469 USA; 2 Interdisciplinary Graduate Studies, College of Arts and Sciences, University of Dayton, Dayton, OH 45469 USA; 3 Premedical Program, University of Dayton, Dayton, OH 45469 USA; 4 The Integrative Science and Engineering Center, University of Dayton, Dayton, OH 45469 USA; 5 Center for Genomic Advocacy (TCGA), Indiana State University, Terre Haute, IN USA

**Keywords:** Apoptosis, Alzheimer's disease

## Abstract

Alzheimer’s disease (AD), a progressive neurodegenerative disorder, manifests as accumulation of amyloid-beta-42 (Aβ42) plaques and intracellular accumulation of neurofibrillary tangles (NFTs) that results in microtubule destabilization. Targeted expression of human Aβ42 (*GMR* > *Aβ42*) in developing *Drosophila* eye retinal neurons results in Aβ42 plaque(s) and mimics AD-like extensive neurodegeneration. However, there remains a gap in our understanding of the underlying mechanism(s) for Aβ42-mediated neurodegeneration. To address this gap in information, we conducted a forward genetic screen, and identified N-acetyltransferase 9 (Mnat9) as a genetic modifier of GMR > Aβ42 neurodegenerative phenotype. Mnat9 is known to stabilize microtubules by inhibiting c-Jun-N- terminal kinase (JNK) signaling. We found that gain-of-function of *Mnat9* rescues *GMR* > *Aβ42* mediated neurodegenerative phenotype whereas loss-of-function of *Mnat9* exhibits the converse phenotype of enhanced neurodegeneration. Here, we propose a new neuroprotective function of Mnat9 in downregulating the JNK signaling pathway to ameliorate Aβ42-mediated neurodegeneration, which is independent of its acetylation activity. Transgenic flies expressing human NAT9 (hNAT9), also suppresses Aβ42-mediated neurodegeneration thereby suggesting functional conservation in the interaction of fly Mnat9 or hNAT9 with JNK-mediated neurodegeneration. These studies add to the repertoire of molecular mechanisms that mediate cell death response following accumulation of Aβ42 and may provide new avenues for targeting neurodegeneration.

## Introduction

Alzheimer’s disease (AD) is an age-related progressive neurodegenerative disease that manifests as neuronal cell death, cognitive impairment(s) and memory loss, with no cure to-date [1–3]. The hallmarks of AD are accumulation of amyloid plaques and intracellular accumulation of neurofibrillary tangles (NFTs). Improper cleavage of the amyloid precursor protein (APP) results in hydrophobic amyloid-beta-42 (Aβ42) monomers that aggregate to form extracellular amyloid plaques [1, 4]. According to the amyloid cascade hypothesis, accumulation of such amyloid plaques and NFTs results in other biochemical changes like oxidative stress, synaptic dysfunction etc. and eventually leads to neurodegeneration [4–7]. Neuronal cell death may occur due to aberrant activation of signaling pathways [8–10]. The mechanism(s) underlying the disease etiology and its progression are yet to be fully understood. Since genetic machinery is conserved across organisms, several animal model systems were developed to understand AD pathophysiology and its underlying mechanism(s) [10–14]. We have previously developed an AD model in *Drosophila melanogaster* (*a.k.a* fruit flies) [8, 10, 15].

*Drosophila* is a highly versatile and genetically tractable model that shares significant conservation of cell signaling pathways and disease related genes with humans [16–19]. The rich repository of molecular genetic tools makes *Drosophila* suitable for studying human disease and performing genome-wide screens [10, 18, 20–23]. The *Drosophila* eye develops from an eye-antennal imaginal disc housed inside the larva [17, 24–27], and has been extensively used to model neurodegenerative disorders [8, 23, 28–30]. The adult eye is a highly organized structure, which makes it easy to study effects of genetic manipulations and screen large sample sizes [10, 17, 18, 22].

We employed the Gal4/UAS system to spatio-temporally target the expression of the human Aβ42 transgene [31] in differentiating retinal neurons using the *GMR-Gal4* driver [32]. Overexpression of human Aβ42 (*GMR* > *Aβ42*), results in a highly reduced, glazed eye phenotype due to accumulation of amyloid plaques. These plaques trigger neuronal cell death and mimic AD like neuropathology [8, 20, 29, 30]. In human patients, cell-based assays and other animal model systems for AD, accumulation of Aβ42 plaques triggers cell death due to increased activity of the evolutionarily conserved c-Jun N-terminal Kinase (JNK) signaling pathway [8, 29, 33–39]. The JNK pathway has been implicated in several cell biological processes like cell proliferation, cell death and cell survival [40]. Activation of JNK or stress activated protein kinases, the members of the highly conserved mitogen-activated protein kinase (MAPK) superfamily, triggers cell death [41]. This pathway is activated when the ligand Eiger, the fly homolog of tumor necrosis factor (TNF), binds to TNF receptors such as Wengen and Grindelwald [40, 42, 43]. Activation of the TNF receptors transmits the signal downstream through a conserved cascade that includes Tak1 (TGF- β-activating kinase 1); a JNK kinase kinase (JNKKK), Hemipterous (Hep; a JNK kinase), and Basket (Bsk; a Jun kinase). Activation of Bsk by phosphorylation in turn activates the downstream transcription factor *Drosophila* Jun related antigen (Jra or dJun) [44]. A functional readout for JNK signaling activation is the relative expression of the target gene *puckered (puc)*. The *puc* encodes a dual specificity phosphatase, and forms a negative feedback loop to downregulate JNK activity [29, 40]. During eye development, JNK signaling activation can trigger caspase-dependent as well as caspase-independent cell death mechanisms [45, 46]. However, our understanding of how these mechanisms impact the neurodegenerative phenotypes caused by Aβ42 accumulation is far from complete.

Using an unbiased genetic approach, we conducted a forward genetic screen to identify modifiers of the *GMR* > *Aβ42* phenotypes in the eye [23, 29]. This screening approach essentially looks for modification of a phenotype – either its enhancement or suppression, and is used to identify genes that likely interact with Aβ42 and may contribute to its effects on neurodegeneration [29, 34]. Thus, identifying genetic modifiers may generate insights into the signaling and molecular mechanisms associated with *GMR* > *Aβ42* phenotypes in the eye. Recently, we screened additional candidate genes using the forward genetic screening platform, and identified a *Drosophila* microtubule-associated N-acetyltransferase (NAT) named *Mnat9* as a genetic modifier of Aβ42-mediated neurodegeneration in the *Drosophila* eye. Nearly 80% of eukaryotic proteins undergo N-α-acetylation, a process where acetyl groups are transferred from acetyl-CoA to the N-terminus. This initial protein modification [47] is mediated through different kinds of N-acetyltransferases (NATs) [48]. NATs are members of a superfamily of enzymes where eight different NAT complexes composed of one or more subunits have been identified including NatA- NatH [48]. N-α-acetylation by NATs mediates multiple biological roles including but not restricted to protein folding, degradation, subcellular localization, and post-translational ER import control [47]. In flies, microtubule-associated protein NAT9 (Mnat9) acetylates the N-terminus of alpha- and beta-Tubulin, subunits of microtubules in vitro [49]. During development, Mnat9 is involved in maintaining microtubule stability to regulate multiple signaling pathways [49, 50]. Previous research shows that overexpression (gain-of-function) of *Mnat9* downregulates the evolutionarily conserved c-Jun N-terminal Kinase (JNK) signaling pathway [49].

Here, we show a new neuroprotective role of Mnat9 in ameliorating the Aβ42-mediated neurodegeneration phenotype in *Drosophila* eye. Gain-of-function of *Mnat9* in the developing *Drosophila* eye ameliorates this phenotype. Whereas the loss-of-function of *Mnat9* enhances the reduced eye phenotype caused by Aβ42 accumulation. Mnat9’s acetylation function is not required for its role in suppressing the neurodegenerative phenotype. However, Mnat9’s neuroprotective role in stabilizing microtubules is mediated through downregulation of the JNK-signaling pathway. Here we demonstrate that upregulation of Mnat9 rescues Aβ42 mediated neurodegeneration by downregulating JNK signaling in the retinal neurons. Furthermore, human NAT9 (hNAT9) can also rescue the Aβ42-mediated neurodegeneration phenotype in *Drosophila* eye suggesting that this neuroprotective function may be conserved.

## Materials and methods

### Stocks

The fly stocks used in this study are *GMR-Gal4* (BL8605), UAS-*Mnat9*-HA, UAS-*Mnat9*^RNAi^, UAS-*hNAT9* [49, 50]*,* UAS*-Mnat9 [AcDel]*, UAS*-Mnat9* [*AAA*], UAS-*hNAT9* [*AAA*] [49], *hep*^Act^/*CyO; TM3 Sb/TM6B,Tb* [51], *Sco/CyO*; UAS-*jun*^aspv7^/*TM3 Sb* [52], *Sco/CyO*; UAS-*bsk*^DN^/*TM3 Sb* [53], *Sco/CyO;* UAS*-puc/TM3 Sb* [45], *Sco/CyO*; *puc*^E69^/*TM6B, Tb* [45] and UAS-*Aβ42* [8, 54] that are listed in Flybase (http://flybase.bio.indiana.edu), Bloomington Stock center and Vienna *Drosophila* Resource Center. The UAS*-Aβ42* transgenic flies were generated by microinjecting a bi-cistronic UAS-construct where two tandem copies of human amyloid - β1-42 (Aβ42) fused to signal peptide for secretion were cloned [8, 54, 55].

### Genetic crosses

We employed a Gal4/ UAS targeted expression system in this study [31]. All Gal4/UAS crosses were maintained at 25 °C; the adult flies were maintained at 25 °C, while the egg-laying were transferred to 29 °C for further growth. The *UAS*-*Aβ42* transgene was expressed under the control of *GMR-Gal4* driver [32] (*GMR-Gal4* > UAS*-Aβ42*), which drives expression in differentiating retinal neurons of the eye imaginal disc and pupal retina. We confirmed that *GMR-Gal4* > UAS-*Aβ42* (*GMR* > *Aβ42*) larvae grown at 29 °C exhibit a stronger neurodegenerative phenotype with 100% penetrance [8]. All *Mnat9*^*RNAi*^ crosses co-expressed with UAS-*dicer2* were crossed to the Gal4 driver to obtain strong RNA interference effects.

### Adult Eye Imaging

We prepared the adult flies (after eclosion) for imaging by freezing them at −20 °C for approximately two hours followed by mounting the flies on a dissection needle. Adult flies of similar age from both sexes were used for adult imaging. The needle was embedded in a clay putty on a glass slide to position the fly horizontally to get lateral or dorsal view of the fly eye/head. We took adult eye images on the Axiomager.Z1 Zeiss Apotome using optical Z-sectioning function [33, 56]. The final images were generated by compiling individual stacks from the Z section using the extended depth of focus function of Axiovision software version 4.6.3.

### Frequency of eye phenotype

For each genetic cross, three independent sets of two hundred flies were checked (200 × 3 = 600) for calculating the frequency of each eye phenotype(s). The eye phenotypes were categorized as no-eye, reduced-eye, reduced-eye with necrotic spots and rescue of neurodegenerative phenotype. Graphs were plotted in GraphPad Prism.

### Quantitative analyses of severity score of eye degenerative phenotype

We examined the eye phenotypes from 200 flies per genotype and scored them according to the following criteria where: “No-eye” was assigned to category 6, 80% eye degeneration was assigned to category 5, 60–80% eye degeneration was assigned to category 4, 40–60% eye degeneration was assigned to category 3, 20–40% eye degeneration was assigned to category 2, 0–20% eye degeneration was assigned to category 1 and wild-type was assigned to category 0. Comparisons were made using non-Parametric: Mann–Whitney t-Test and graphs were plotted in GraphPad Prism 8.3.1.

### Quantitative analyses of area of the eye

The adult eye images were analyzed in ImageJ software and region of interest (ROI) was drawn along the perimeter of the adult eye shown as white dotted line in figures. We measured the surface area of the eye by using ImageJ software and plotted graph in GraphPad Prism 8.3.1.

### Immunohistochemistry

Eye-antennal imaginal discs were dissected from the third instar larvae in cold 1X phosphate buffered saline (PBS), fixed in 4% paraformaldehyde in 1X PBS for 20 min, and stained using a standardized protocol [57]. Primary antibodies used were rat anti-Embryonic Lethal Abnormal Vision (ELAV) (1:100; Developmental Studies Hybridoma Bank, DSHB, Catalogue number #7E8A10), mouse anti-Discs-large (Dlg) (1:100; DSHB, Catalogue number #4F3), mouse anti-acetylated Tubulin (1:100; DSHB, Catalogue number #12G10), mouse anti-Chaoptin (24B10) (1:100; DSHB, Catalogue number #24B10) [58], mouse anti-6E10 (1:100; Covance, Catalogue number #SIG-39320) and rabbit Mnat9 (1:100; a gift from Dr. Kwang-Wook Choi) [49]. Secondary antibodies (Jackson Laboratory) used were goat anti-rat IgG conjugated with Cy5 (1:250; Catalogue number #112-175-143), donkey anti-mouse IgG conjugated with Cy3 (1:250; Catalogue number #715-165-150) and donkey anti-rabbit IgG conjugated with Cy3 (1:250; Catalogue number #711-165-152). We mounted the tissues in antifading agent Vectashield (Vector Laboratories). The immunofluorescent images were captured at 20X magnification using Olympus Fluoview 3000 Laser Scanning Confocal Microscope [59]. All final figures were prepared using Adobe Photoshop software.

### Detection of cell death

Apoptosis was detected by a cell death detection kit from Roche Diagnostics using terminal deoxynucleotidyl transferase dUTP nick end labeling (TUNEL) assay. TUNEL assay labels DNA breakage by adding fluorescently labeled nucleotides to free 3′-OH DNA ends in a template-independent manner using terminal deoxynucleotidyl transferase (TdT) enzyme. The fluorescein labels (TMR red) incorporated in nucleotide polymers can be detected by fluorescence microscopy [60, 61]. The TUNEL assay was performed according to the standardized protocol [46, 62]. The TUNEL positive cells were counted from five sets of imaginal discs of each genotype and were used for the statistical analysis using Microsoft Excel 2013 [62]. The *p*-values were calculated using Student’s *t* test, and the error bars represent standard error of mean (SEM).

### DHE staining

The third instar larval eye-antennal imaginal discs were dissected in cold 1X Schneider’s *Drosophila* medium (Gibco, Catalogue number #21720024). The samples were incubated in Dihydroethidium (DHE, Life Technologies Catalogue number # D11347) dye solution [(1:300) in 1XPBS] [63, 64] for 5 min and were washed three times with cold 1X PBS. DHE is oxidized by superoxide radical to form 2-hydroxyethidium which intercalates with DNA and provides signal at 550 nm in cells where ROS is produced [64–66]. The eye discs were then mounted on a slide and were immediately imaged live on Olympus Fluoview 3000, a Laser Scanning Confocal microscope [59]. All final figures were prepared using Adobe Photoshop software. The number of ROS puncta were quantified from five sets of imaginal discs per genotype by using automated quantification method [64]. The Interactive H watershed plugin of Fiji/ ImageJ free software was used for automated quantification and the statistical analysis was performed using Microsoft Excel [64]. The p-values were calculated using Student’s *t* test, and the error bars represent standard error of mean (SEM) *p-value < 0.05, **p-value < 0.01, ***p-value < 0.001.

### Real-time quantitative polymerase chain reaction

Real-time quantitative polymerase chain reaction (RT-qPCR) was performed according to the standardized protocol [67, 68]. Total RNA was extracted in 500 μl of TRIzol Reagent (Thermo Fisher, Catalogue Number # 15596926) from twenty pairs of third instar larvae eye-antennal imaginal discs (*n* = 40), which were dissected from *GMR-Gal4, GMR* > *Aβ42, GMR* *>* *Mnat9, GMR* > *Aβ42* *+* *Mnat9, GMR* *>* *Mnat9*^*RNAi*^*, GMR* > *Aβ42* *+* *Mnat9*^*RNAi*^ backgrounds. The quality of isolated RNA was determined by checking the A260/A280 ratio using a Nanodrop 2000 spectrophotometer (Thermo Scientific) and confirmed that the ratio was greater than 2. cDNA was produced from total RNA through RT-PCR using the first-strand cDNA synthesis kit (GE healthcare, Catalogue number # 27926101). qPCR was performed using iQ™ SYBR Green Supermix (Bio-Rad) and Bio- Rad iCycler (Bio-Rad) following the kit’s protocol for 25 μl. Primers used for *jun* are: (fwd: CCAACCGTCCGAAACTATGT; rev: CCGGCGGCTATTCTGATTATTA). The expression level of Glyceraldehyde 3-phosphate dehydrogenase (GAPDH) was used as an internal control to normalize the results (fwd: CAATGGATTTGGTCGCATCG; rev: CCGTTGACCACCAGGAAACC). The fold change was calculated relative to the expression level of the respective controls, using the delta delta C_T_ method.

### Western blotting

Protein samples were prepared from (*n* = 25) adult fly heads in RIPA lysis buffer following a standardized protocol [69, 70]. The protein samples were loaded in a 10% gel, and transferred onto a nitrocellulose membrane. The membrane was washed, blocked in 5% w/v BSA in 1X TBST and incubated overnight at 4 °C with primary antibody rabbit Phospho-SAPK/JNK (1:1000) (Cell Signaling Thr183/Tyr185) (81E11) antibody and mouse anti-α-Tubulin antibody (1:12000) (SIGMA, Catalogue number. # T5168) diluted in 5% w/v BSA in 1X TBST. This was followed by 1 h incubation with secondary antibody: horseradish peroxidase conjugated goat anti-rabbit IgG-HRP (1:5000) and goat anti-mouse IgG-HRP (1:5000) (Santa Cruz Biotechnology, Catalogue number: Sc-2005). The signal was detected using Super Signal West Dura Extended Duration Substrate (ThermoFisher Scientific, Catalogue number: #34076). Images were captured using the LI-COR Odyssey Fc imaging system. Relative pJNK levels were then statistically quantified and normalized by using LI-COR Image Studio lite 5.2 software and graph was plotted in GraphPad Prism 8.3.1.

### Eclosion assay

Eclosion assays are used to screen the effect of genetic backgrounds on eclosion of flies. We collected eggs on a grape plate from *Elav-Gal4* (control), *Elav* > *Aβ42*, *Elav* > *Aβ42* + *Mnat9* and *Elav* > *Aβ42* + *Mnat9*^*RNAi*^. We seeded the first instar larvae (30 in each set) from a synchronous culture in each vial. 270 larvae (9 sets of 30 larvae) were counted for each cross. The larvae were allowed to develop to adult hood, and eclosion rate counted. All unhatched pupae were also counted. The graph was plotted in GraphPad Prism 8.3.1.

## Results

### *Mnat9* is a genetic modifier of Aβ42-mediated neurodegeneration

**Fig. 1:**
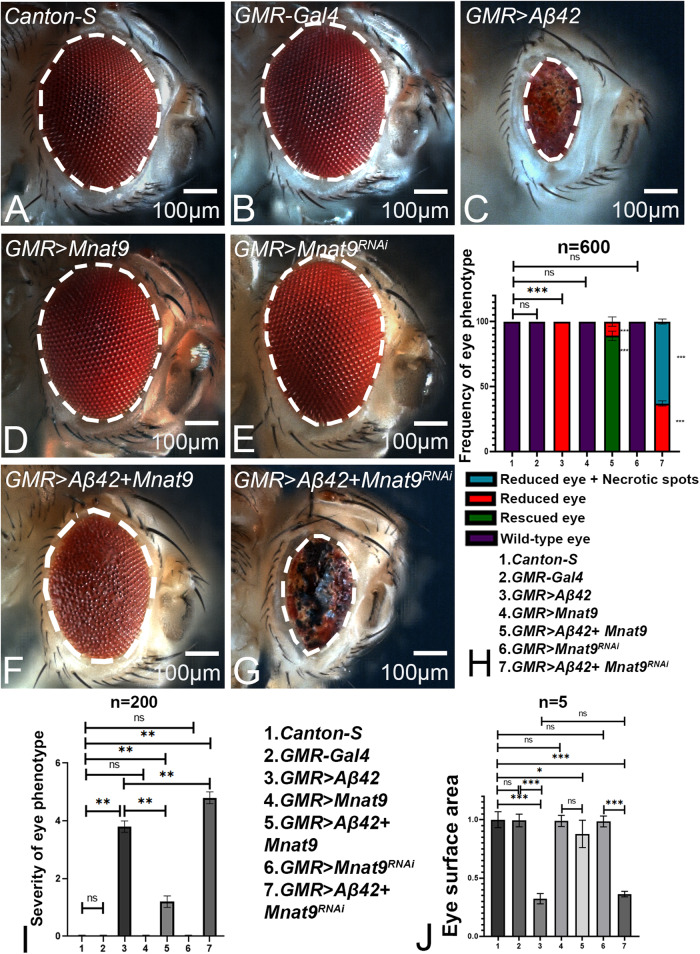
*Mnat9* is a modifier of Aβ42-mediated neurodegeneration. Adult eye of (**A**) Wild type, (**B**) *GMR-Gal4*, (**D**) *GMR>Mnat9* and (**E**) *GMR>Mnat9*^RNAi^ serve as controls. **C** Overexpression of human Aβ42 in the developing eye (*GMR* > *Aβ42*) results in a highly reduced glazed adult eye phenotype. **F** Gain-of-function of *Mnat9* in *GMR* > *Aβ42* (*GMR* > *Aβ42*+*Mnat9*) background results in significant rescue whereas (**G**) downregulating *Mnat9* (*GMR* > *Aβ42* + *Mnat9*^RNAi^) enhances *GMR* > *Aβ42* neurodegenerative phenotype. **H** Bar graph shows frequency of eye phenotype(s). Six hundred flies were counted for each genotype (1. *Canton-S*, 2. *GMR-Gal4*, 3. *GMR* > *Aβ42*, 4. *GMR* *>* *Mnat9*, 5. *GMR* > *Aβ42* *+* *Mnat9*, 6. *GMR* > *Mnat9*^*RNAi*^ and 7. *GMR* > *Aβ42* *+* *Mnat9*^*RNAi*^) for calculating the frequency of eye phenotype(s). Statistical analysis was performed using the Student’s *t* test for independent samples. **I** Quantitative analyses of severity score of neurodegenerative phenotype(s) in eye. Flies from each genotype were randomly selected for scoring according to criteria described in the methods section. Comparisons were made using non-Parametric Mann–Whitney *t* Test. **J** Quantitative analyses of area of the eye. The surface area of the eye (within white dotted line) was calculated using Image J. Statistical analysis was performed using the Student’s *t* test for independent samples. The surface area is significantly rescued in *GMR* > *Aβ42* + *Mnat9* (*n* = 5; *p* = 0.000045) as compared to *GMR* > *Aβ42* whereas it is significantly reduced in *GMR* > *Aβ42* *+* *Mnat9*^*RNAi*^ (*n* = 5; *p* = 0.00336). Error bars show standard error of mean (mean ± SEM), and symbols above the error bar signify as ***p-value < 0.001, **p-value < 0.01, *p-value < 0.05, and not significant (n.s.) *p*-value > 0.05 respectively. Scale bar = 100 μm.

The wild-type adult compound eye is comprised of 600–800-unit eyes (Fig. 1A, H–J), and *GMR-Gal4* exhibits near normal eye morphology (Fig. 1B, H–J). Targeted expression of human amyloid-beta 42 using a *GMR-Gal4* driver (*GMR* > *Aβ42*) exhibits a strong neurodegenerative phenotype characterized by the generation of a highly reduced eye with disorganized and fused ommatidia with 100% penetrance (*n* = 600) [8] (Fig. 1C, H–J). Using a forward genetic screen strategy [23], we identified *Mnat9* as a genetic modifier of *GMR* > *Aβ42* neurodegenerative phenotype. We classified the eye phenotypes into four categories for statistical analyses: reduced plus necrotic eye (Fig. 1G), reduced eye (like *GMR* > *Aβ42* eye, Fig. 1C), rescue (increase in size from *GMR* > *Aβ42* eye, Fig. 1F) and wild-type eye (Fig. 1A, B). Gain-of-function of *Mnat9* in *GMR* > *Aβ42* background (*GMR* > *Aβ42* + *Mnat9*) significantly rescues the Aβ42-mediated neurodegenerative phenotype as observed in the adult eyes (*n* = 600, 516/600 = 86%) (Fig. 1F, H–J). Overexpression of *Mnat9* alone (*GMR* > *Mnat9*), which serves as a control, exhibits a near normal adult eye phenotype (Fig. 1D). Loss-of-function of *Mnat9* in *GMR* > *Aβ42* background by using RNA interference approach (*GMR* > *Aβ42* + *Mnat9*^RNAi^) enhances the neurodegenerative phenotype with necrotic spots (*n* = 600, 372/600 = 62%) (Fig. 1G, H–J) as compared to the *GMR* > *Aβ42* alone (Fig. 1C, H–J). The control *GMR>Mnat9*^RNAi^ alone does not show any neurodegenerative phenotype (Fig. 1E). Moreover, gain-of-function of *Mnat9* in the *GMR* > *Aβ42* flies significantly suppressed the eye degenerative phenotype (Fig. 1I) and increased the eye surface (Fig. 1J). These results validate our previous findings from the forward genetic screen that show *Mnat9* as a genetic modifier of Aβ42-mediated neurodegeneration in the *Drosophila* eye. A comparison of eye phenotypes between males and females for each experimental and control groups did not reveal any significant differences (Supplementary Fig. 1A–M).

### Mnat9 is expressed ubiquitously in the developing eye

**Fig. 2:**
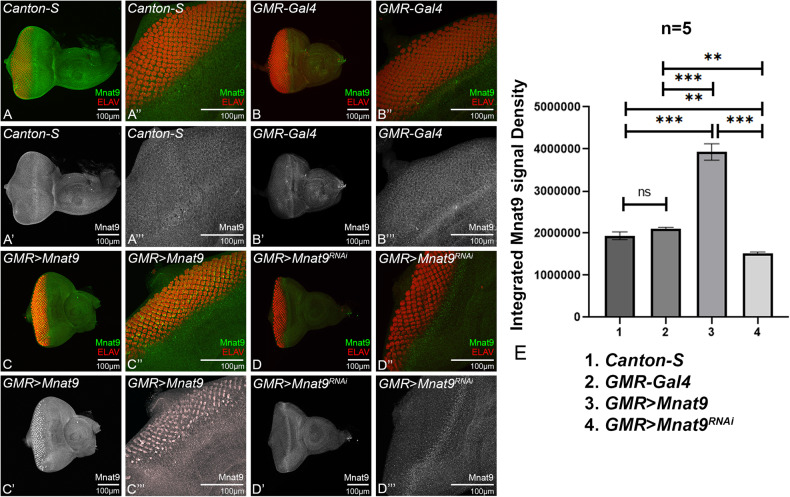
Mnat9 is expressed ubiquitously in the developing eye. The third instar larval eye imaginal discs were stained with the pro-neural marker embryonic lethal abnormal vision (ELAV; shown in red) and an anti-Mnat9 antibody (green or gray). (**A**–**D** 20× magnification, A’’–D’’ 60× magnification) Eye discs showing expression of Mnat9 and ELAV, whereas (A’–D’ 20× magnification, A’’’–D’’’ 60× magnification) shows expression of Mnat9 alone (gray). A’–D’ Eye discs of all (**A**) *Canton S*, (**B**) *GMR-Gal4*, (**C**) *GMR* > *Mnat9*, (**D**) *GMR* > *Mnat9*^*RNAi*^. **E** Bar graph shows the comparison of intensity of Mnat9 expression (Mnat9 levels) in controls and experimental eye discs. Mnat9 expression was statistically quantified by Fiji/ ImageJ software within the region of interest (GMR domain). Number of samples, *N* = 5 was used per genotype for all calculations and the graph show data for 1. *Canton-S*, 2. *GMR-Gal4*, 3. *GMR* > *Mnat9*, 4. *GMR* > *Mnat9*^*RNAi*^. Statistical analysis was performed using the Student’s *t* test for independent samples. Error bars show standard error of mean (mean ± SEM), and symbols above the error bar signify as ***p-value < 0.001, **p-value < 0.01, *p-value < 0.05, and not significant (n.s.) *p*-value > 0.05 respectively. The orientation of all imaginal discs is identical with posterior to the left and dorsal up. Scale bar = 100 μm.

We employed anti-Mnat9 antibody to study expression of Mnat9 in larval eye disc and brain. The signal intensity of Mnat9 expression was statistically quantified within the region of interest (ROI) marked by yellow dotted line (Fig. 2A’–D’, A’’’–D’’’) by using ImageJ software (Fig. 2A–E). We confirmed that Mnat9 is ubiquitously expressed in the wild-type and *GMR-GAL4* eye discs (Fig. 2A’, B’, A’’’, B’’’). Furthermore, overexpression of *Mnat9* (*GMR* *>* *Mant9*) resulted in significant increase in Mnat9 expression (Fig. 2C, C’’’, E) whereas downregulation of *Mnat9* (*GMR* *>* *Mnat9*^RNAi^) resulted in reduction of Mnat9 expression in the GMR domain (Fig. 2D, D”’, E).

### *Mnat9* does not affect the Aβ42 levels

**Fig. 3:**
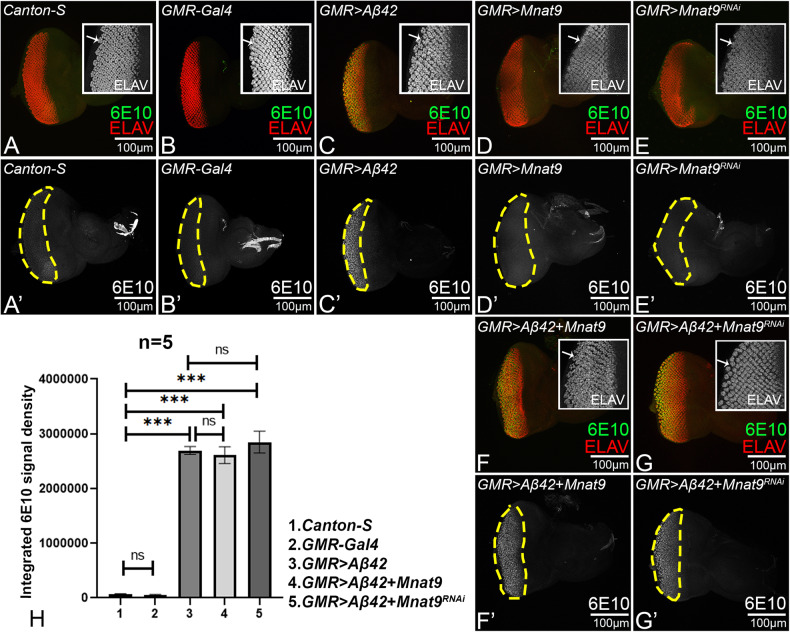
*Mnat9* does not affect Aβ42 levels. The third instar larval eye imaginal discs were stained with the pro-neural marker Embryonic Lethal Abnormal Vision (ELAV; shown in red) to mark the nuclei of retinal neurons and 6E10, an anti-human Aβ42 antibody (green or gray). **A**–**G** Eye discs show 6E10 and ELAV staining. Inset marked by white boundary shows zoom images ELAV and (A’–G’) 6E10 expression in gray scale mode. **A**, **B**, A’-B’ Wild-type and *GMR-Gal4* controls do not show 6E10 staining. **C**, C’ *GMR* > *Aβ42* eye discs exhibits robust Aβ42 expression marked by 6E10. The controls (**D**, D’) *GMR* > *Mnat9* and (**E**, E’) *GMR* *>* *Mnat9*^*RNAi*^ show absence of Aβ42 based on 6E10 staining. Level of Aβ42 in (**F**, F’) *GMR* > *Aβ42* + *Mnat9* (**G**, G’) *GMR* > *Aβ42* *+* *Mnat9*^RNAi^ background. Note that there is no significant change in Aβ42 levels in *GMR* > *Aβ42* + *Mnat9* and *GMR* > *Aβ42* *+* *Mnat9*^RNAi^ as compared to *GMR* > *Aβ42* eye discs. **H** Bar graph shows quantification of the 6E10 levels, which were statistically quantified within the region of interest, marked by yellow dotted line, using Fiji/ ImageJ software. Number of samples, *N* = 5 was used per genotype for the calculation (1. *Canton-S*, 2. *GMR-Gal4*, 3. *GMR* > *Aβ42*, 4. *GMR* > *Aβ42* *+* *Mnat9*, 5. *GMR* > *Aβ42* *+* *Mnat9*^RNAi^). Statistical analysis was performed using the Student’s *t* test for independent samples. *GMR* > *Aβ42* *+* *Mnat9* (*n* = 5; *p* = 0.765) and *GMR* > *Aβ42* *+* *Mnat9*^RNAi^ (*n* = 5; *p* = 0.167) have no significant change in Aβ42 levels as compared to *GMR* > *Aβ42* eye discs. Error bars show standard error of mean (mean ± SEM), and symbols above the error bar signify as ***p-value < 0.001, **p-value < 0.01, *p-value < 0.05, and not significant (n.s.) *p*-value > 0.05 respectively. The orientation of all imaginal discs is identical with posterior to the left and dorsal up. Scale bar = 100 μm.

A possible mechanism for Mnat9 mediated modification of *GMR* > *Aβ42* phenotype can be due to the reduction of Aβ42 levels. To confirm this hypothesis, we employed 6E10 antibody staining in *GMR* > *Aβ42* and GMR > Aβ42+*Mnat9* eye imaginal disc [8, 29, 33]. The signal intensity of 6E10 was statistically quantified within the region of interest (ROI) marked by yellow dotted line (Fig. 3A’–G’) using the ImageJ software (Fig. 3A–H). As expected, no 6E10 signal was recorded in controls like Canton-S (Fig. 3A, A’, H) and *GMR-Gal4* (Fig. 3B, B’, H) that did not express human Aβ42. In comparison, strong accumulation of Aβ42 plaques were seen in *GMR* > *Aβ42* eye discs, which results in a progressive neurodegenerative phenotype with disorganized and increased spaces in the photoreceptors mostly at the posterior margin of the eye disc (Fig. 3C, C’, H) [8]. Additionally, the other negative controls: *GMR* *>* *Mnat9*, and *GMR* *>* *Mnat9*^RNAi^ did not show Aβ42 plaques accumulation (Fig. 3D, D’, E, E’). Based on signal intensity, co-expression of *Mnat9* with *GMR* > *Aβ42* (*GMR* > *Aβ42* *+* *Mnat9*) did not show any significant change in Aβ42 levels (Fig. 3F, F’, H) as compared to *GMR* > *Aβ42* eye discs (Fig. 3C, C’, H). Similarly, loss-of-function of *Mnat9* in *GMR* > *Aβ42* (*GMR* > *Aβ42* *+* *Mnat9*^RNAi^) also did not show any significant change in Aβ42 plaque levels (Fig. 3G, G’, H) as compared to *GMR* > *Aβ42* eye discs (Fig. 3C, C’, H). These findings suggests that modulation of *Mnat9* likely affects signals downstream of the Aβ42 plaque accumulation.

### *Mnat9* prevents axonal targeting defects seen in *GMR* > *Aβ42*

**Fig. 4:**
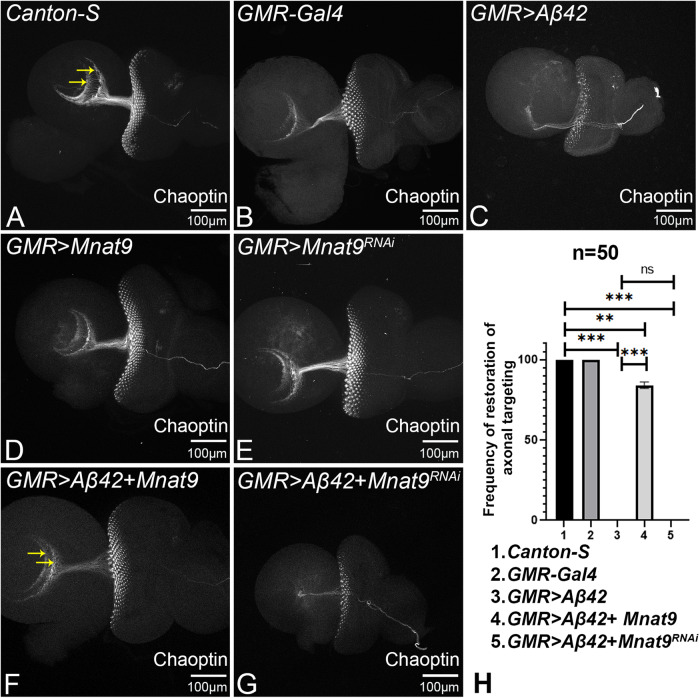
Gain-of-function of *Mnat9* restores axonal targeting seen in *GMR* > *Aβ42* background. The retinal axons of (**A**) *Canton-S* (wild-type), (**B**) *GMR-Gal4*, (**D**) *GMR>Mnat9* and (**E**) *GMR* *>* *Mnat9*^RNAi^, marked by Chaoptin (24B10) staining, which innervates the two layers of the brain, marked by two yellow arrows. Note that impaired axonal targeting from retina seen in (**C**) *GMR* > *Aβ42*, is restored by (**F**) gain-of-function of *Mnat9* (*GMR* > *Aβ42*+*Mnat9*) whereas (**G**) loss-of-function of *Mnat9* (*GMR* > *Aβ42* + *Mnat9*^RNAi^) disrupts the axonal targeting. **J** Bar graph shows frequency of axonal targeting phenotype. Sample size was 50 for each genotype (1. *Canton-S*, 2. *GMR-Gal4*, 3. *GMR* > *Aβ42*, 4. *GMR* > *Aβ42* *+* *Mnat9*, 5. *GMR* > *Aβ42* *+* *Mnat9*^RNAi^). Statistical analysis was performed using the Student’s *t* test for independent samples. *GMR* > *Aβ42* *+* *Mnat9* significantly restores the axonal targeting (*n* = 5; *p* = 0.000019) whereas *GMR* > *Aβ42* *+* *Mnat9*^RNAi^ disrupt the axonal targeting (*n* = 5; *p* = 0.3465) as compared to *GMR* > *Aβ42*. Error bars show the standard error of mean (mean ± SEM), and symbols above the error bar signify as ***p-value < 0.001, **p-value < 0.01, *p-value < 0.05, and not significant (n.s.) *p*-value > 0.05 respectively. The orientation of all imaginal discs is identical with posterior to the left and dorsal up. Magnification of all eye imaginal discs is 20×. Scale bar = 100 μm.

The reduced eye phenotype in *GMR* > *Aβ42* flies is accompanied with disruption in axonal guidance and targeting [8, 23, 28, 33, 71]. Each ommatidium in the *Drosophila* eye is comprised of eight photoreceptors (R1-R8). The axons from these photoreceptors bundle innervate different regions in the *Drosophila* brain, for example, R1-R6 innervate the lamina whereas R7-R8 extend into medulla of the brain [72, 73]. Chaoptin (m24B10) serves as a reliable marker to study retinal axons and their projections to the brain (Fig. 4A) [58]. To check effects on axonal targeting, we counted a total of *n* = 50 eye discs per genotype and recorded the frequency of eye discs showing rescue of axonal targeting in comparison to *GMR* > *Aβ42* (Fig. 4A–H). *GMR-Gal4* eye discs show similar axonal projections in the brain as seen in *Canton-S* (wild-type) eye discs (Fig. 4B, H). However, in the *GMR* > *Aβ42* eye imaginal disc, the retinal axonal targeting gets impaired as evident from the highly reduced axonal tract and disorganization in axonal targeting (Fig. 4C, H) [33, 71]. These neuronal defects likely contribute to AD phenotypes. Gain-of-function of *Mnat9* alone in GMR domain (*GMR* *>* *Mnat9*) and loss-of-function of *Mnat9* (*GMR* *>* *Mnat9*^RNAi^), which also serve as controls and exhibit wild-type axonal targeting (Fig. 4D, E). Interestingly, overexpression of *Mnat9* with Aβ42 (*GMR* > *Aβ42* *+* *Mnat9*) significantly restores the observed axonal targeting defects to near wild-type in ~82% (*n* = 50, 41/50 = 82%) of the imaginal discs stained and imaged (Fig. 4F, H) as compared to *GMR* > *Aβ42* eye discs (Fig. 4C, H). However, loss-of-function of *Mnat9* in *GMR* > *Aβ42* background (*GMR* > *Aβ42+Mnat9*^RNAi^) shows impaired axonal targeting similar to *GMR* > *Aβ42* eye imaginal discs in 100% (*n* = 50) of the observed imaginal discs (Fig. 4G, H). Therefore, Mnat9 may prevent the axonal targeting defects seen in retinal neurons of *GMR* > *Aβ42* flies.

### *Mnat9* can block Aβ42 mediated cell death in *Drosophila* eye

**Fig. 5:**
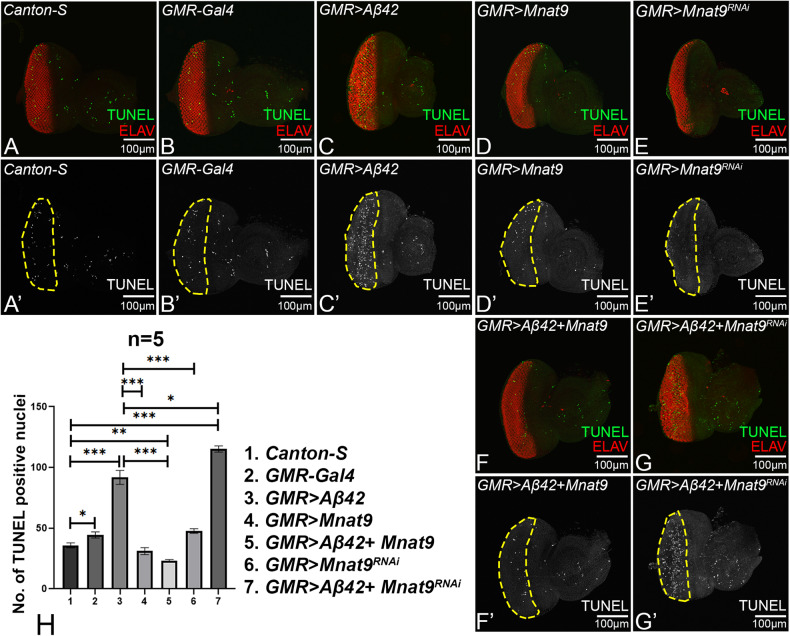
Gain-of-function of *Mnat9* downregulates cell death. **A**–**G** Eye discs stained for proneural marker ELAV (red) to mark the nuclei of retinal neurons and TUNEL (green) to mark dying nuclei. A’-G’ Split channel showing TUNEL only expression. **A**, A’ *Canton-S* and **B**, B’ *GMR-Gal4*, **C**, C’ *GMR* > *Aβ42*, **D**, D’ *GMR>Mnat9*, **E**, E’ *GMR* *>* *Mnat9*^RNAi^, **F**, F’ *GMR* > *Aβ42* *+* *Mnat9* and **G**, G’ *GMR* > *Aβ42* *+* *Mnat9*^RNAi^. Note that controls (**A**, **B**, **D**, **E**) exhibit some random TUNEL positive nuclei. However, gain-of-function of *Mnat9* (**F**, F') *GMR* > *Aβ42* *+* *Mnat9* exhibits significant reduction in TUNEL positive dying cell as compared to (**C**, C’) *GMR* > *Aβ42*. **G**, G’ Downregulation of *Mnat9* shows the converse phenotype. **H** The TUNEL positive nuclei were statistically quantified within the yellow dotted line- the region of interest (ROI). Number of samples = 5 was used per genotype for the calculation (1. *Canton-S*, 2. *GMR-Gal4*, 3. *GMR* > *Aβ42*, 4. *GMR* *>* *Mnat9*, 5. *GMR* > *Aβ42* + *Mnat9*, 6. *GMR* *>* *Mnat9*^RNAi^ and 7. *GMR* > *Aβ42* + *Mnat9*^RNAi^). Statistical analysis was performed using the Student’s *t* test for independent samples. *GMR* > *Aβ42* *+* *Mnat9* exhibits significant reduction in TUNEL positive nuclei as compared to *GMR* > *Aβ42* (*n* = 5; *p* = 0.00049) whereas *GMR* > *Aβ42* + *Mnat9*^RNAi^ shows slight increase in TUNEL positive nuclei as compared to *GMR* > *Aβ42* (*n* = 5; *p* = 0.3587). Error bars show the standard error of mean (mean ± SEM), and symbols above the error bar signify as ***p-value < 0.001, **p-value < 0.01, *p-value < 0.05, and not significant (n.s.) *p*-value > 0.05 respectively. The orientation of all imaginal discs is identical with posterior to the left and dorsal up. Scale bar = 100 μm.

Next, we tested if *Mnat9* can modulate retinal neuron cell death observed in the *GMR* > *Aβ42* background. We employed terminal deoxynucleotidyl transferase dUTP nick end labeling (TUNEL) staining to mark the nuclei of dying cells [62]. TUNEL positive cells were counted from five imaginal discs per genotype within the region of interest (ROI), marked by yellow dotted line, and were used for statistical analysis (Fig. 5A-H, A’-G’). A few cells undergo cell death in wild-type (Fig. 5A, A’, H) and GMR-Gal4 (Fig. 5B, B’, H) eye imaginal discs. However, the *GMR* > *Aβ42* eye imaginal discs show nearly a two-fold increase in the number of TUNEL positive nuclei (Fig. 5C, C’, H). The other control *GMR>Mnat9* (Fig. 5D, D’), shows a similar number of TUNEL positive nuclei as wild type. However, *GMR>Mnat9*^RNAi^ eye discs exhibit an increase in the number of TUNEL positive nuclei as compared to wild-type (Fig. 5E, E’). Gain-of-function of *Mnat9* in the *GMR* > *Aβ42* background (*GMR* > *Aβ42+Mnat9*) (Fig. 5F, F’, H) results in a nearly six-fold reduction in the number of dying cells as compared to *GMR* > *Aβ42* (Fig. 5C, C’, H). Loss-of-function of *Mnat9* in *GMR* > *Aβ42* background (*GMR* > *Aβ42+ Mnat9*^RNAi^) (Fig. 5G, G’, H) results in a significant increase in the number of dying nuclei as compared to the wild-type (Fig. 5A, A', H). Thus, TUNEL data suggests that *Mnat9* might downregulate the cell death caused by Aβ42-accumulation. It has been previously reported that Aβ42 aggregate triggers production of reactive oxygen species (ROS) [64, 74].

### Overexpression of *Mnat9* downregulates ROS production

**Fig. 6:**
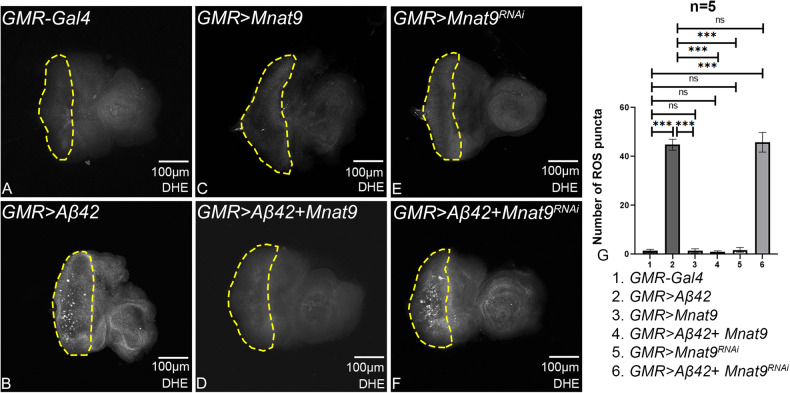
Modulation of *Mnat9* in *GMR* > *Aβ42* downregulates ROS production. Dihydroethidium (DHE) is employed to detect ROS produced in cells. **A** *GMR-Gal4* shows minimal level of ROS puncta. **B** *GMR* > *Aβ42* shows elevated levels of ROS puncta. **C** *GMR* *>* *Mnat9* and **E** *GMR* *>* *Mnat9*^RNAi^ serve as controls, show a very few ROS puncta. **D** *GMR* > *Aβ42* + *Mnat9* results in significant reduction in the ROS production as compared to *GMR* > *Aβ42*. **F** *GMR* > *Aβ42* *+* *Mnat9*^RNAi^ results in the increase in ROS production. **A**–**F** ROS puncta were counted within yellow dotted line, the region of interest (ROI), for the statistical analysis. We quantified ROS puncta in photoreceptor cells of five eye imaginal discs per genotype (*n* = 5) (1. *GMR-Gal4*, 2. *GMR* > *Aβ42*, 3. *GMR>Mnat9*, 4. *GMR* > *Aβ42* + *Mnat9*, 5. *GMR* *>* *Mnat9*^RNAi^ and 6. *GMR* > *Aβ42* + *Mnat9*^RNAi^). Statistical analysis was performed using Student’s *t* test for independent samples. *GMR* > *Aβ42* *+* *Mnat9* exhibits significant reduction in ROS puncta as compared to *GMR* > *Aβ42* (*n* = 5; *p* = 0.00000053) whereas *GMR* > *Aβ42* *+* *Mnat9*^RNAi^ (*n* = 5; *p* = 0.8) show slightly increased ROS puncta as compared to *GMR* > *Aβ42*. Error bars show the standard error of mean (mean ± SEM), and symbols above the error bar signify as ***p-value < 0.001, **p-value < 0.01, *p-value < 0.05, and not significant (ns), *p*-value > 0.05 respectively. The orientation of all imaginal discs is identical with posterior to the left and dorsal up. Scale bar = 100 μm.

Accumulation of amyloid plaques triggers oxidative stress in neurons resulting in an imbalance in the generation of reactive oxygen species (ROS) and antioxidant defense mechanism [8, 64, 75, 76]. Induction of ROS leads to oxidative modification of biomolecules in postmitotic neurons that are associated with AD pathology [64, 75, 76]. Hence, we measured the ROS levels using dihydroethidium (DHE) staining in eye-antennal imaginal discs when *Mnat9* levels were modulated in the background of *GMR* > *Aβ42* flies. The ROS puncta were counted within ROI (yellow dotted line) from five imaginal discs per genotype and were used for statistical analyses (Fig. 6A–G). Overexpression of Aβ42 (*GMR* > *Aβ42;* Fig. 6B, G) results in a significant increase in ROS production as compared to minimal ROS levels seen in *GMR-Gal4* background (Fig. 6A, G). Interestingly, overexpression of *Mnat9* in the *GMR* > *Aβ42* background (*GMR* > *Aβ42* + *Mnat9*; Fig. 6D, G) shows reduction in levels of ROS signal as compared to the *GMR* > *Aβ42* background (Fig. 6B, G). Loss-of-function of *Mant9* in the *GMR* > *Aβ42* background (*GMR* > *Aβ42* *+* *Mnat9*^RNAi^; Fig. 6F, G) shows slightly increased ROS levels as compared to the *GMR* > *Aβ42* (Fig. 6B, G). Hence, high levels of *Mnat9* can downregulate ROS levels in *GMR* > *Aβ42* flies.

### Overexpression of *Mnat9* suppresses the mortality of Aβ42 expressing flies

**Fig. 7:**
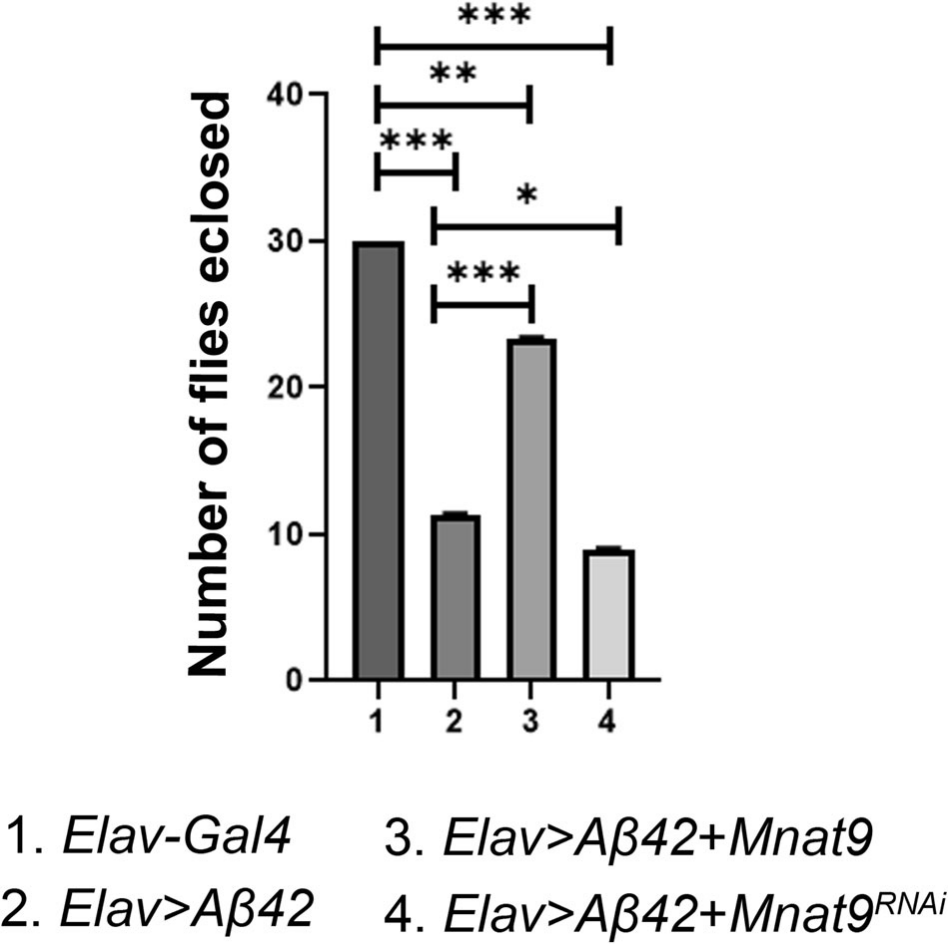
Overexpression of *Mnat9* reduces the mortality rate of *Elav* > *Aβ42* flies. The bar graph represents the number of flies eclosed. We compared the number of flies eclosed in 1. *Elav-Gal4* (control), 2. *Elav* > *Aβ42*, 3. *Elav* > *Aβ42*+*Mnat9* and 4. *Elav* > *Aβ42*+*Mnat9*^RNAi^ background and validated that overexpression of *Mnat9* in the *Elav* > *Aβ42* background rescues the *Elav* > *Aβ42* mortality rate. We counted 270 flies in three independent biological sets from each background and plotted on a graph in GraphPad Prism 8.3.1. Error bars show the standard error of mean (mean ± SEM), and symbols above the error bar signify as ***p-value < 0.001, **p-value < 0.01, *p-value < 0.05, and not significant (ns), *p*-value > 0.05 respectively.

In AD flies with overexpressed *Mnat9*, we observed an increase in the survival of retinal neurons. Therefore, to corroborate these observations, we modulated levels of *Mnat9* in the fly neurons by using *Elav-Gal4* driver that drives expression of transgene in fly neurons [33]. Misexpression of human Aβ42 using *Elav-Gal4* (*Elav* > *Aβ42)* resulted in high mortality rate as only 40% (*n* = 270) of the flies could hatch out and survive whereas remaining 60% population were arrested as larvae or pupae. In contrast, all wild-type flies eclosed and did not show any lethality (Fig. 7, *n* = 270, 100%). We analyzed mortality rate when *Mnat9* was modulated in an *Elav* > *Aβ42* background. Overexpression of *Mnat9* significantly increased the survival rate of *Elav* > *Aβ42* flies (*Elav* > *Aβ42* *+* *Mnat9*; Fig. 7, *n* = 270) as 76% of flies hatched. On the other hand, when *Mnat9* was downregulated (*Elav* > *Aβ42* *+* *Mnat9*^RNAi^; Fig. 7, *n* = 270) only 30% of the flies eclosed and 70% flies failed to hatch out due to pupal and larval lethality suggesting an enhancement of the mortality rate compared to *Elav* > *Aβ42* flies.

### Acetyltransferase domain of *Mnat9* is not required to rescue neurodegeneration

**Fig. 8:**
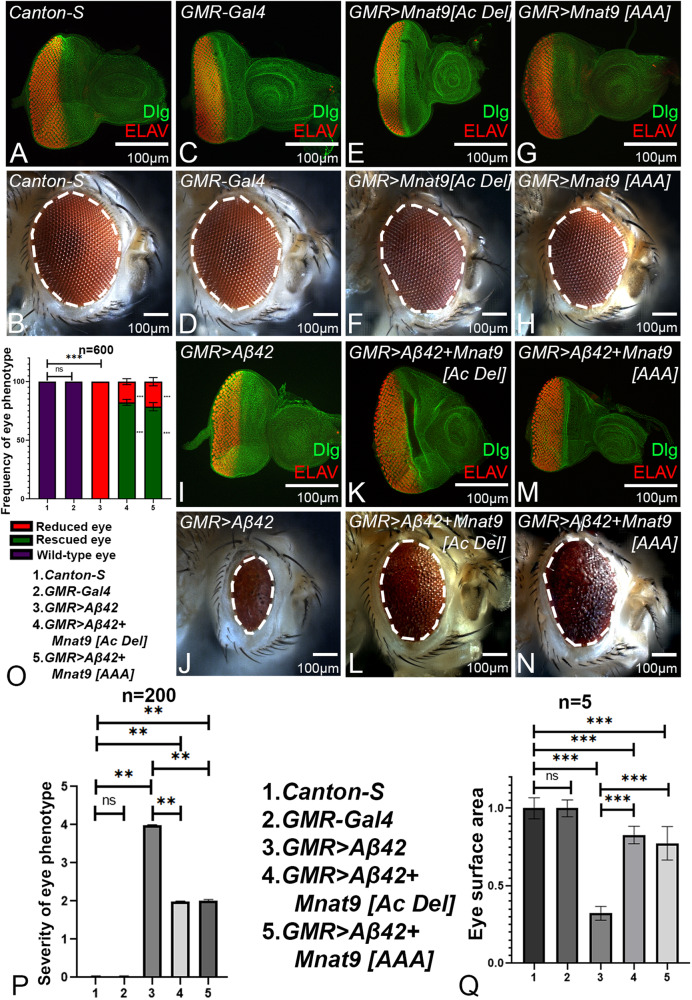
Acetylation activity of Mnat9 is not required for its neuroprotective function. Eye imaginal discs were stained with membrane specific marker, Discs large (Dlg; green), and a pan neural marker ELAV (red) to mark the nuclei of retinal neurons. Eye Imaginal disc and adult eye (**A**, **B**) *Canton-S* (wild-type), (**C**, **D**) *GMR-Gal4*, (**E**, **F**) *GMR* *>* *Mnat9* [*Ac Del*] and (**G**, **H**) *GMR* *>* *Mnat9* [*AAA*] eye served as control. **I** Overexpression of human Aβ42 in the developing eye imaginal disc (*GMR* > *Aβ42*) leads to (**J**) severe, reduced adult eye phenotype. Overexpression of acetylated defective *Mnat9* in the *GMR* > *Aβ42* background (**K**, **L**) *GMR* > *Aβ42* + *Mnat9* [*Ac Del*] and (**M**, **N**) *GMR* > *Aβ42* + *Mnat9* [*AAA*] result in significant rescue in eye disc and adult eye as compared to the *GMR* > *Aβ42*. **O** Bar graph shows frequency of eye phenotype(s). Six hundred flies were counted for calculating the frequency for each genotype (1. *Canton-S*, 2. *GMR-Gal4*, 3. *GMR* > *Aβ42*, 4. *GMR* > *Aβ42* *+* *Mnat9* [*Ac Del*], 5. *GMR* > *Aβ42* *+* *Mnat9* [*AAA*]). Statistical analysis was performed using the Student’s *t* test for independent samples. **P** Quantitative analyses of severity score of eye degenerative phenotype(s). Flies from each genotype were randomly selected for scoring according to criteria described in the methods section. Comparisons were made using non-Parametric: Mann–Whitney *t* Test. **Q** Quantitative analyses of area of the eye. The surface area of the eye (within white dotted line) was calculated using Image J. Statistical analysis was performed using the Student’s *t* test for independent samples. The surface area of the eye is significantly rescued in *GMR* > *Aβ42* + *Mnat9* [*Ac Del*] (*n* = 5; *p* = 7.73E−08) and *GMR* > *Aβ42* + *Mnat9* [*AAA*] (*n* = 5; *p* = 6.75E−05) as compared to *GMR* > *Aβ42*. Error bars show the standard error of mean (mean ± SEM), and symbols above the error bar signify as ***p-value < 0.001, **p-value < 0.01, *p-value < 0.05, and not significant (n.s.) *p*-value > 0.05 respectively. Scale bar = 100 μm.

NATs N-α-acetylation function plays a role in several cell biological processes like protein folding, degradation, subcellular localization, and post-translational ER import control [47]. Mnat9 has a N-acetyltransferase domain that can acetylate N-terminal peptides of α- and β-Tubulin in vitro [49]. To test if acetyl transferase activity of *Mnat9* is required for its neuroprotective function in the *GMR* > *Aβ42* background, we used transgenic flies where the functional acetylation domain of *Mnat9* was mutated. The Mnat9 reference protein has a well-conserved acetyl-CoA binding motif (Q/RxxGxG/A) that is critical for its N-terminal acetylation activity. The motif was mutated in two constructs: *Mnat9* [*AAA*] has alanine substitutions AxxAxA in the RxxGxG acetyl-CoA binding site and *Mnat9* [*AcDel*] has a deletion of six amino acids in the RxxGxG acetyl-CoA binding site (RGKGFG) respectively [49]. The *Canton-S* and *GMR-Gal4* control flies have normal eye discs (Fig. 8A, C) and adult eye phenotypes (Fig. 8B, D, O–Q). The other controls like *GMR>Mnat9* [*AcDel*], and *GMR>Mnat9* [*AAA*] also exhibit near wild-type eye discs (Fig. 8E, G) and adult eye phenotypes (Fig. 8F, H). *GMR* > *Aβ42* flies exhibits a progressive neurodegenerative phenotype with disorganized and increased spaces in the photoreceptors mostly at the posterior margin of the eye disc (Fig. 8I), which gets further aggravated in the adult eye (Figs. 1L, S, 8J, O–Q) [8]. Overexpression of *Mnat9* [*AcDel*] construct in the background of *GMR* > *Aβ42* (*GMR* > *Aβ42* *+* *Mnat9 [Ac Del]*) (*n* = 600, 494/600 = 82.3%) (Fig. 8K, L, O–Q) exhibits significant rescue as compared to *GMR* > *Aβ42* alone (Fig. 8I, J, O–Q). Overexpression of *Mnat9* [*AAA*] construct in the background of *GMR* > *Aβ42* (*GMR* > *Aβ42* + *Mnat9* [*AAA*]) (*n* = 600, 472/600 = 78.6%) (Fig. 8M–Q) exhibit significant rescue as compared to *GMR* > *Aβ42* alone (Fig. 8I, J, O–Q). The complete loss of acetylated domain *GMR* > *Aβ42* + *Mnat9* [*AcDel*] (Fig. 8K, L, O–Q) shows a stronger rescue than alanine substitution constructs: *GMR* > *Aβ42* *+* *Mnat9* [*AAA*] (Fig. 8M–Q). We also calculated frequency of phenotypes (Fig. 8O), severity of eye phenotype (Fig. 8P) and surface area of the eye (Fig. 8Q) in the backgrounds discussed above. We also immuno-stained the eye discs with anti-acetylated Tubulin antibody and observed that the acetylated Tubulin level did not change dramatically in controls versus *GMR* > *Aβ42* background as well as when *Mnat9* levels were modulated (Supplementary Fig. 2). These results also suggest that acetylation activity is not required. Taken together, these data support that the acetylation domain is not required for Mnat9’s neuroprotective function in the context of Aβ42-mediated neurodegeneration.

### *Mnat9* suppresses JNK activity

**Fig. 9:**
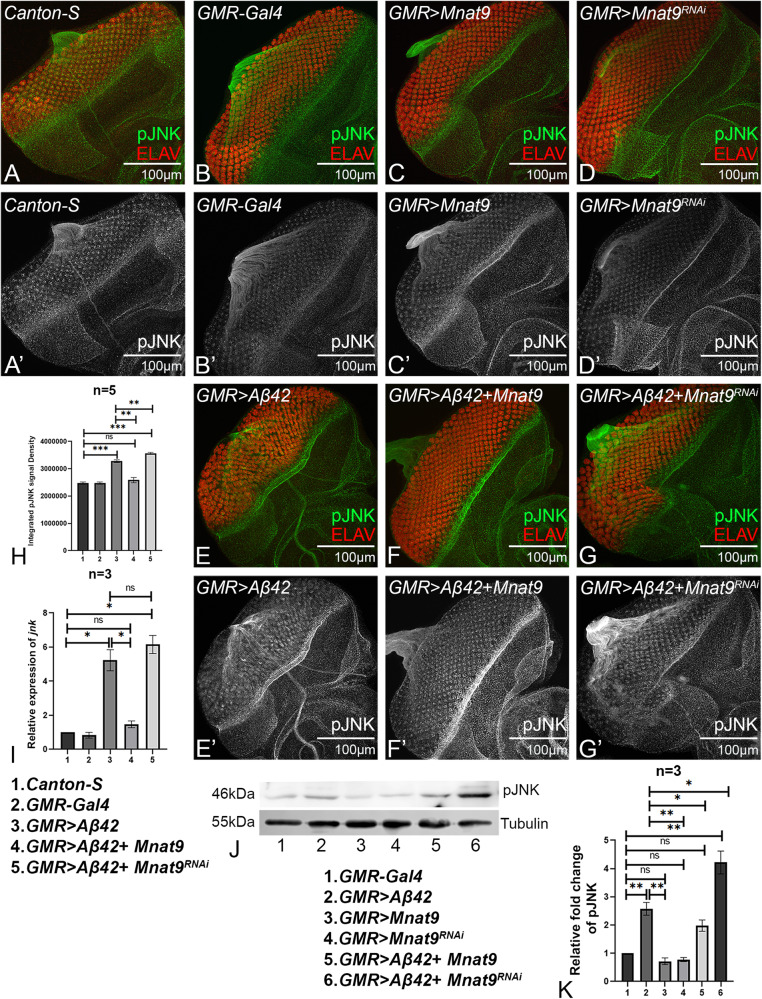
*Mnat9* regulates pJNK levels in the developing eye. Eye discs were stained with pan neuronal marker ELAV (red) to mark the nuclei of retinal neurons and pJNK (green) marks the phosphorylated JNK (shows the activation of JNK pathway). A’–G’ Split channel shows pJNK level in gray scale mode. **A**, A’, **B**, B’ *Canton-S* and *GMR-Gal4*, serve as controls, show expression of pJNK. **C**, C’ *GMR* *>* *Mnat9* and (**D**, D’) *GMR* *>* *Mnat9*^RNAi^ serve as other controls. **E**, E’ The eye discs of *GMR* > *Aβ42* shows significant increase in pJNK levels. **F**, F’ Gain-of-function of *Mnat9* in the *GMR* > *Aβ42* background (*GMR* > *Aβ42* + *Mnat9*) shows significant downregulation in the pJNK levels as compared to *GMR* > *Aβ42*. Whereas (**G**, G’) reduced levels of *Mnat9* in the *GMR* > *Aβ42* background (*GMR* > *Aβ42* + *Mnat9*^RNAi^) shows slight increase in the pJNK levels as compared to *GMR* > *Aβ42*. **H** Bar graph shows the integrated density of pJNK levels. The pJNK levels were statistically quantified within the region of interest by Fiji/ ImageJ software. Number of samples (*n* = 5) per genotype was used for the calculation (1. *Canton-S*, 2. *GMR-Gal4*, 3. *GMR* > *Aβ42*, 4. *GMR* > *Aβ42* + *Mnat9*, 5. *GMR* > *Aβ42* + *Mnat9*^RNAi^). Statistical analysis was performed using the Student’s *t* test for independent samples. *GMR* > *Aβ42* + *Mnat9* shows significant reduction in the pJNK levels (*n* = 5; *p* = 0.001) whereas *GMR* > *Aβ42* + *Mnat9*^RNAi^ shows slight increase in the pJNK levels (*n* = 5; *p* = 0.005) as compared to *GMR* > *Aβ42*. **I** Relative expression of *jnk* at the transcriptional level using quantitative PCR (qRT-PCR) in genotypes (1. *Canton-S*, 2. *GMR-Gal4*, 3. *GMR* > *Aβ42*, 4. *GMR* > *Aβ42* + *Mnat9*, 5. *GMR* > *Aβ42* + *Mnat9*^RNAi^). Triplicate was used for the calculation. Statistical analysis was performed using Student’s *t* test for independent samples. The relative *jnk* transcript expression was significantly reduced in *GMR* > *Aβ42* + *Mnat9* (*n* = 3; *p* = 0.017) whereas slightly increased in *GMR* > *Aβ42* + *Mnat9*^RNAi^ (*n* = 3; *p* = 0.317) as compared to *GMR* > *Aβ42*. **J** The western blot shows the expression levels of the pJNK protein in the *Drosophila* eye. The samples were loaded in the following sequence: Lane 1: *GMR-Gal4*, Lane 2: *GMR* > *Aβ42*, Lane 3: *GMR* *>* *Mnat9*, Lane 4: *GMR* *>* *Mnat9*^RNAi^, Lane 5: *GMR* > *Aβ42* + *Mnat9*, Lane 6: GMR > Aβ42 + *Mnat9*^RNAi^. Alpha Tubulin is used as a loading control. The molecular weight of pJNK is 46 kDa, and alpha Tubulin is 55 kDa. The samples were treated with anti-pJNK antibody, and anti-α Tubulin antibody. **K** Bar graph representing a relative pJNK level, signal intensity of the bands, which clearly demonstrates that when *Mnat9* is misexpressed in the *GMR* > *Aβ42* background, pJNK levels are downregulated (*n* = 3; *p* = 0.04) whereas downregulation of *Mnat9* upregulates pJNK levels (*n* = 3, *p* = 0.024). Triplicate was used for the calculation. Statistical analysis was performed using Student’s *t* test for independent samples. Error bars show the standard error of mean (mean ± SEM), and symbols above the error bar signify as ***p-value < 0.001, **p-value < 0.01, *p-value < 0.05, and not significant (n.s.) *p*-value > 0.05 respectively. The orientation of all imaginal discs is identical with posterior to the left and dorsal up. Scale bar = 100 μm.

Earlier it has been shown that JNK signaling is activated in both conditions such as accumulation of amyloid plaques [8, 29, 30, 33] and when Mnat9 levels are downregulated [49]. We therefore investigated if Mnat9 modulates JNK signaling pathway to rescue *GMR* > *Aβ42* phenotype. Activation of JNK signaling triggers a cascade of kinases that ultimately triggers cell death. We checked the levels of JNK activation by quantifying levels of phospho-JNK by immunohistochemistry, and western blot. We quantified the intensity of pJNK levels by ImageJ software posterior to the morphogenetic furrow (Fig. 9A–H, A’-G’) [8, 30]. A significant increase in pJNK levels was seen in *GMR* > *Aβ42* as compared to the wild-type eye disc (Fig. 9A, A’, B, B’, E, E’, H) [8]. However, in comparison to *GMR* > *Aβ42*, pJNK levels were reduced in *GMR* > *Aβ42* + *Mnat9* eye discs (Fig. 9F, F’, H). In contrast, pJNK levels were significantly increased when *Mnat9* was knocked down in the *GMR* > *Aβ42* background (*GMR* > *Aβ42* + *Mnat9*^RNAi^) (Fig. 9G, G’, H) as compared to the wild-type (Fig. 6A, A’, B, B’, H). Using real time PCR, we further validated that *jnk* transcript levels were significantly reduced (~2.5-fold) in *GMR* > *Aβ42* + *Mnat9* as compared to *GMR* > *Aβ42* (Fig. 9I). Moreover, *jnk* transcript levels were slightly increased when *Mnat9* was knockdown in *GMR* > *Aβ42* background (*GMR* > *Aβ42* + *Mnat9*^RNAi^) as compared to *GMR* > *Aβ4*2 (Fig. 6I). We also validated the pJNK protein levels by western blot semi-quantitative analysis and observed a significant increase in pJNK levels when *Mnat9* was knockdown in *GMR* > *Aβ42* flies (Fig. 9J, K). pJNK levels were significantly decreased in *GMR* > *Aβ42* + *Mnat9* as compared to *GMR* > *Aβ42*. These evidences suggest that *Mnat9* ameliorates Aβ42- mediated neurodegeneration by suppressing the JNK activity.

### *Mnat9* downregulates JNK signaling to rescue Aβ42-mediated neurodegeneration

**Fig. 10:**
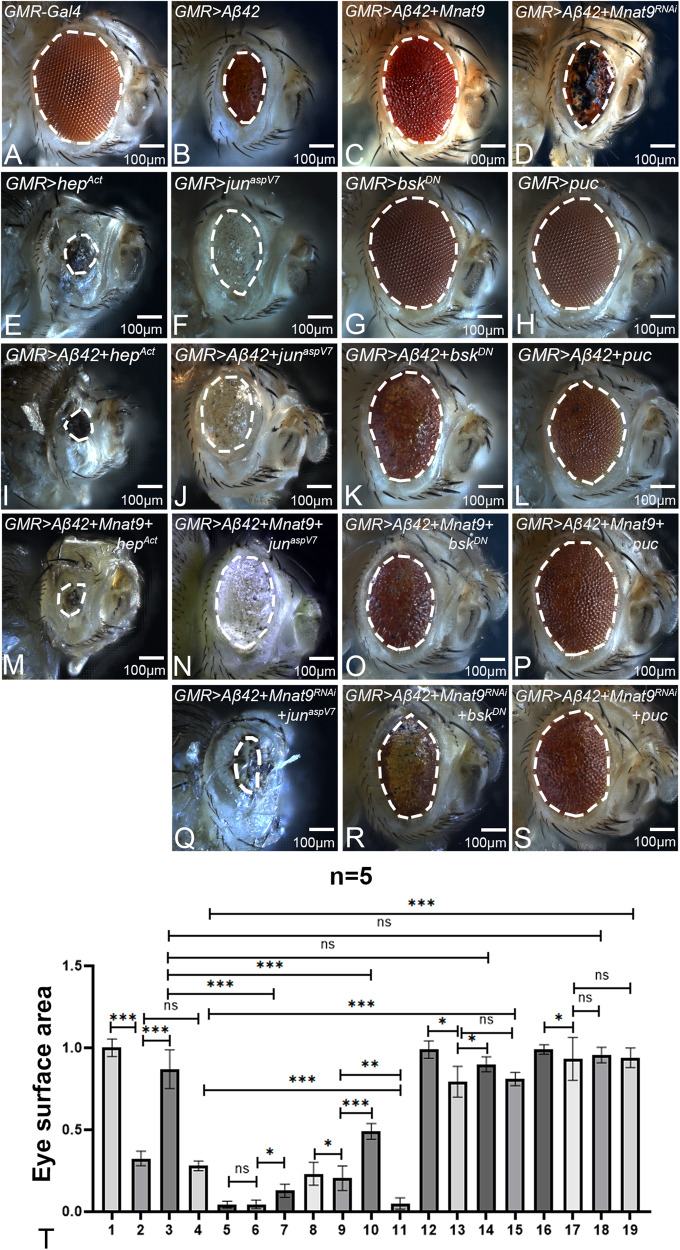
Mnat9 regulates JNK signaling pathway in developing eye. **A** *GMR-Gal4* serves as control. **B** *GMR* > *Aβ42* adult eye is highly reduced due to neurodegeneration. **C** Gain-of-function of *Mnat9* in the background of *GMR* > *Aβ42* (*GMR* > *Aβ42*+*Mnat9*) results in significant rescue in the adult eye as compared to the *GMR* > *Aβ42* reduced eye phenotype. **D** However, downregulating *Mnat9* levels in *GMR* > *Aβ42* flies (*GMR* > *Aβ42*+*Mnat9*^RNAi^) enhances *GMR* > *Aβ42* neurodegenerative phenotype. **E** *GMR>hep*^Act^ and **F** *GMR>jun*^aspV7^ results in a severe neurodegenerative phenotype in the eye. Whereas (**G**) *GMR>bsk*^DN^ and (**H**) *GMR>puc*, do not affect the size of the adult eye, serve as controls. **I** *GMR* > *Aβ42* + *hep*^Act^ and **J** *GMR* > *Aβ42* + *jun*^aspV7^ results in a dramatic neurodegenerative eye phenotype in the adult fly. However, **K** *GMR* > *Aβ42* + *bsk*^DN^ and **L** *GMR* > *Aβ42* + *puc* results in a significant rescue as compared to *GMR* > *Aβ42* adult eye. Gain-of-function of *Mnat9* in the background of *GMR* > *Aβ42* resulted in a slight rescue when JNK signaling pathway was upregulated (**M**) *GMR* > *Aβ42* *+* *Mnat9* + *hep*^Act^ and (**N**) *GMR* > *Aβ42* *+* *Mnat9* + *jun*^aspv7^. Downregulation of the JNK pathway in (**O**) *GMR* > *Aβ42+Mnat9*+*bsk*^DN^ and (**P**) *GMR* > *Aβ42* *+* *Mnat9* + *puc* showed a significant rescue eye phenotype. **Q** Loss-of-function of *Mnat9* in the background of *GMR* > *Aβ42* resulted in not eclosed flies with worsen eye phenotype when the JNK signaling pathway was upregulated: *GMR* > *Aβ42* *+* *Mnat9*^RNAi^ + *jun*^aspv7^. Downregulation of the JNK pathway in (**R**) *GMR* > *Aβ42* *+* *Mnat9*^RNAi^ + *bsk*^DN^ and (**S**) *GMR* > *Aβ42* *+* *Mnat9*^RNAi^ + *puc* showed a significant rescue eye phenotype as compared to *GMR* > *Aβ42* *+* *Mnat9*^RNAi^. **T** Quantitative analyses of area of the eye. The surface area of the eye (within white dotted line) was calculated using ImageJ. Five flies were used for calculating the eye surface area for each genotype (1. *GMR-Gal4*, 2. *GMR* > *Aβ42*, 3. *GMR* > *Aβ42* + *Mnat9*, 4. *GMR* > *Aβ42* *+* *Mnat9*^RNAi^, 5. *GMR* *>* *hep*^Act^ 6. *GMR* > *Aβ42* + *hep*^Act^, 7. *GMR* > *Aβ42* + *Mnat9* + *hep*^Act^, 8. *GMR* *>* *jun*^aspV7^, 9. *GMR* > *Aβ42*+ *jun*^aspV7^, 10. *GMR* > *Aβ42+ Mnat9*+ *jun*^aspV7^, 11. *GMR* > *Aβ42* *+* *Mnat9*^RNAi^ + *jun*^aspV7^, 12. *GMR* *>* *bsk*^*DN*^, 13. *GMR* > *Aβ42* + *bsk*^DN^, 14. *GMR* > *Aβ42* + *Mnat9* + *bsk*^DN^, 15. *GMR* > *Aβ42* *+* *Mnat9*^RNAi^ + *bsk*^DN^, 16. *GMR* *>* *puc*, 17. *GMR* > *Aβ42* + *puc*, 18. *GMR* > *Aβ42* + *Mnat9* + *puc*, 19. *GMR* > *Aβ42* + *Mnat9*^RNAi^ + *puc*). Statistical analysis was performed using the Student’s *t* test for independent samples. Error bars show the standard error of mean (mean ± SEM), and symbols above the error bar signify as ***p-value < 0.001, **p-value < 0.01, *p-value < 0.05, and not significant (n.s.) *p*-value > 0.05 respectively. Scale bar = 100 μm.

Previous studies from our lab have shown that blocking the JNK pathway rescues Aβ42-mediated neurodegeneration [8, 29, 30, 33]. In order to understand if *Mnat9* rescues Aβ42-mediated neurodegeneration by suppressing JNK signaling, levels of JNK pathway members along with gain-of-function of *Mnat9* in *GMR* > *Aβ42* background were analyzed. JNK pathway was activated using *hep*^Act^ and *jun*^aspv7^. The controls, *GMR* *>* *hep*^Act^ (Fig. 10E, T; Supplementary Fig. 3A, B) and GMR > *jun*^aspv7^ (Fig. 10F, T; Supplementary Fig. 3A, B) exhibit cell death in the adult eye as compared to the *GMR-Gal4* control (Fig. 10A, T; Supplementary Fig. 3A, B). Adult eye development was significantly worsened when *hep*^Act^ and *jun*^aspv7^ were overexpressed in the *GMR* > *Aβ42* background [(*GMR* > *Aβ42* + *hep*^Act^) (Fig. 10I, T; Supplementary Fig. 3A, B) and (*GMR* > *Aβ42* + *jun*^aspv7^) (Fig. 10J, T; Supplementary Fig. 3A, B)] respectively. *GMR* > *Aβ42* + *hep*^Act^ (Fig. 10I, T; Supplementary Figs. 3A, B and 5E, E’, P) shows increased cell death as compared to *GMR* > *Aβ42* + *jun*^aspv7^ (Fig. 10J, T; Supplementary Figs. 3A, B and 5F, F’, P). Gain-of-function of *Mnat9* in the background of *GMR* > *Aβ42* *+* *hep*^Act^ (*GMR* > *Aβ42* *+* *hep*^Act^ + *Mnat9*) (Fig. 10M, T; Supplementary Figs. 3A, B and 5I, I’, P) results in significant rescue as compared to *GMR* > *Aβ42* *+* *hep*^Act^ (Fig. 10I, T; Supplementary Figs. 3A, B and 5E, E’, P). Similarly, gain-of-function of *Mnat9* in the background of *GMR* > *Aβ42* *+* *jun*^aspv7^ (*GMR* > *Aβ42* *+* *jun*^aspv7^ + *Mnat9*) (Fig. 10N, T; Supplementary Figs. 3A, B and 5J, J’, P) results in rescue as compared to *GMR* > *Aβ42* *+* *jun*^aspv7^ (Fig. 10J, T; Supplementary Figs. 3A, B and 5F, F’, P). Thus, these findings show that gain-of-function of *Mnat9* rescues Aβ42-mediated neurodegeneration by downregulating JNK signaling pathway. We further tested the downregulation of the JNK signaling pathway by overexpressing the dominant negative *basket* allele (*bsk*^DN^) and *puckered* (*puc*) in our neurodegeneration models. *GMR* *>* *bsk*^DN^ (Fig. 10G, T; Supplementary Fig. 3A, B) and *GMR* *>* *puc* (Fig. 10H, T; Supplementary Fig. 3A, B) served as controls and exhibit near wild-type eyes respectively. Both *GMR* > *Aβ42* *+* *bsk*^DN^ (Fig. 10K, T; Supplementary Figs. 3A, B and 5G, G’, P) and *GMR* > *Aβ42* + *puc* (Fig. 10L, T; Supplementary Figs. 3A, B and 5G, G’, P) show rescue as compared to *GMR* > *Aβ42* (Fig. 10B, T; Supplementary Figs. 3A, B and 5B, B’, P) phenotype. Gain-of-function of *Mnat9* in the background of *GMR* > *Aβ42* *+* *puc* (*GMR* > *Aβ42* *+* *puc* + *Mnat9*) (Fig. 10P, T; Supplementary Figs. 3A, B and 5L, L’, P) results in a significant rescue as compared to GMR > *Aβ42* *+* *bsk*^DN^ + *Mnat9* (Fig. 10O, T; Supplementary Figs. 3A, B and 5K, K’, P). Loss-of-function of *Mnat9* in the background of *GMR* > *Aβ42* *+* *jun*^aspv7^ (*GMR* > *Aβ42* *+* *jun*^aspv7^ + *Mnat9*^RNAi^) (Fig. 10Q, T; Supplementary Figs. 3A, B and 5M, M’, P) results in a severe phenotype with necrotic spots and excessive cell death as compared to *GMR* > *Aβ42* *+* *Mnat9*^RNAi^ (Fig. 10D, T; Supplementary Figs. 3A, B and 5D, D’, P). The downregulation of JNK pathway in *GMR* > *Aβ42* + *Mnat9*^RNAi^ + *bsk*^DN^ (Fig. 10R, T; Supplementary Figs. 3A, B and 5N, N’, P) and *GMR* > *Aβ42* + *Mnat9*^RNAi^ + *puc* (Fig. 10S, T; Supplementary Figs. 3A, B and 5O, O’, P) exhibits a strong rescue and reduced cell death as compared to *GMR* > *Aβ42* *+* *Mnat9*^RNAi^ (Fig. 10D, T; Supplementary Figs. 3A, B and 5D, D’, P). Since *puc* is a transcriptional target of JNK signaling, *puc*-lacZ reporter expression is extensively utilized as a functional read-out of JNK activity [45]. In *GMR-Gal4* eye discs weak expression of *puc*-lacZ is seen in photoreceptor cells (Supplementary Fig. 4A, A’). Other controls like *GMR* *>* *Mnat9* (Supplementary Fig. 4B, B’) and *GMR* *>* *Mnat9*^RNAi^ (Supplementary Fig. 4C, C’) show weak *puc*-lacZ expression in photoreceptor cells as compared to wild-type. We observed a significant increase in *puc*-lacZ expression in *GMR* > *Aβ42* in posterior region of the eye disc as compared to *GMR-Gal4* as seen earlier (Supplementary Fig. 4D, D’) [8]. However, in comparison to *GMR* > *Aβ42*, *puc*-lacZ expression was significantly reduced in *GMR* > *Aβ42* + *Mnat9* (Supplementary Fig. 4E, E’). In contrast, *puc*-lacZ expression was significantly increased when *Mnat9* was knocked-down in *GMR* > *Aβ42* background (*GMR* > *Aβ42* + *Mnat9*^RNAi^) (Supplementary Fig. 4F, F’) as compared to the wild-type. These results suggest that *Mnat9* downregulates the JNK signaling pathway.

### Mnat9 function is conserved in humans

**Fig. 11:**
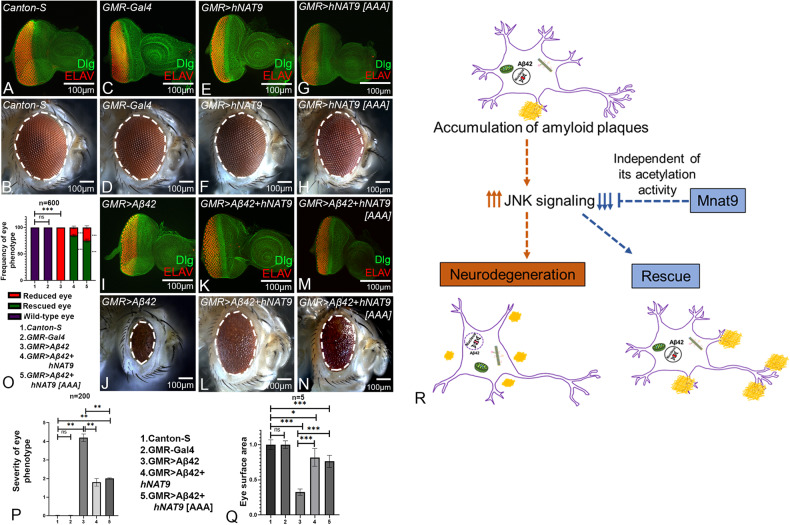
Mnat9 and human hNAT9 exhibit functional conservation. The eye imaginal discs which are stained with membrane specific marker, Discs large (Dlg; green), and a pan neural marker ELAV (red) to mark the nuclei of retinal neurons. **A** *Canton-S* (wild-type) third instar larval eye imaginal disc develops in to (**B**) an adult compound eye. **C**, **D** *GMR-Gal4*, facilitates the expression of target transgenes in the developing eye, and serves as a control. **E**, **F** *GMR* > *hNAT9* and **G**, **H** *GMR* > *hNAT9* [*AAA*] also serve as control. **I** Overexpression of human Aβ42 in the developing eye imaginal disc (*GMR* > *Aβ42*) leads to (**J**) severe, reduced adult eye phenotype. **K** Gain-of-function of *hNAT9* in the background of *GMR* > *Aβ42* (*GMR* > *Aβ42* + *hNAT9*) results in significant rescue in eye disc and (**L**) adult eye as compared to the *GMR* > *Aβ42*. **M**, **N** *GMR* > *Aβ42* + *hNAT9* [*AAA*] shows a significant increase in eye size as compared to the *GMR* > *Aβ42*. **O** Bar graph shows frequency of eye phenotype(s). Six hundred flies were counted for calculating the frequency for each genotype (1. *Canton-S*, 2. *GMR-Gal4*, 3. *GMR* > *Aβ42*, 4. *GMR* > *Aβ42* + *hNAT9*, 5. *GMR* > *Aβ42* + *hNAT9* [*AAA*]). Statistical analysis was performed using the Student’s *t* test for independent samples. **P** Quantitative analyses of severity score of eye degenerative phenotype(s). Flies from each genotype were randomly selected for scoring according to criteria described in the methods section. Comparisons were made using non-Parametric: Mann–Whitney *t* Test. **Q** Quantitative analyses of area of the eye. The surface area of the eye (within white dotted line) was calculated using Image J. Statistical analysis was performed using the Student’s *t* test for independent samples. The surface area of the eye is significantly rescued in *GMR* > *Aβ42* + *hNAT9* (*n* = 5; *p* = 0.000135) and *GMR* > *Aβ42* + *hNAT9* [*AAA*] (*n* = 5; *p* = 0.0000108) as compared to *GMR* > *Aβ42*. Error bars show standard error of mean (mean ± SEM), and symbols above the error bar signify as *** *p*-value < 0.001, **p-value < 0.01, *p-value < 0.05, and not significant (n.s.) *p*-value > 0.05 respectively. Scale bar = 100 μm. **R** Overexpression of Aβ42 in the fly eye (*GMR* > *Aβ42*) results in accumulation of amyloid plaques extracellularly and causes aberrant activation of the JNK pathway resulting in a neurodegenerative adult eye phenotype. Overexpression of *Mnat9* and *hNAT9* in the background of *GMR* > *Aβ42*, ameliorate Aβ42-mediated neurodegeneration by downregulating JNK pathway, independent of its acetylation activity.

We also tested whether human NAT9 (hNAT9), a human homolog of fly Mnat9 that belongs to the GCN5 family [49], can also ameliorate Aβ42-mediated neurodegeneration. In comparison to the eye disc or adult eye from wild-type (Fig. 11A, B, O–Q) or *GMR-Gal4* (Fig. 11C, D, O–Q) controls, *GMR* > *Aβ42* exhibits strong neurodegenerative phenotype (Fig. 11I, J, O–Q). Misexpression of *hNAT9* alone, (*GMR* > *hNAT9*) serves as another control and exhibits near normal eye imaginal disc and adult eye phenotype (Fig. 11E, F). Gain-of-function of *hNAT9* in *GMR* > *Aβ42* background (*GMR* > *Aβ42* + *hNAT9*) significantly rescues the Aβ42-mediated neurodegenerative phenotype as observed in the eye disc and adult eye (*n* = 600, 528/600 = 88%) (Fig. 8K, L, O–Q). In order to test if the acetyl transferase activity of hNAT9 functions in a similar fashion as *Drosophila* Mnat9, and is not required for its neuroprotective function in *GMR* > *Aβ42* background, we used transgenic flies where functional acetylation domain of *hNAT9* was mutated. The motif was mutated in *hNAT9* construct (*hNAT9* [*AAA*]) by substituting alanine AxxAxA in the RxxGxG acetyl-CoA binding site [49]. The control *GMR* > *hNAT9* [AAA] exhibit near wild-type eye discs (Fig. 11G) and adult eye phenotypes (Fig. 11H). Misexpression of *hNAT9* [*AAA*] construct in the background of *GMR* > *Aβ42* (*GMR* > *Aβ42* + *hNAT9* [*AAA*]) (*n* = 600, 450/600 = 75%) (Fig. 11M–Q) exhibit a significant rescue as compared to *GMR* > *Aβ42* alone (Fig. 11I, J, O–Q). We also calculated frequency of phenotypes (Fig. 11O), severity of eye phenotype (Fig. 8P) and the surface area of eye (Fig. 11Q) in various background(s) discussed above. Based on these three parameters, we found that hNAT9 can significantly rescue the neurodegeneration as seen with *Drosophila* Mnat9. Taken together, these three parameters further validate that hNAT9 shows functional conservation to fly Mnat9 in its ability to modify Aβ42- mediated neurodegeneration phenotypes by downregulating the JNK signaling pathway, which is independent of the acetylation function of NAT9 (Fig. 11R).

## Discussion

We identified *Mnat9* as a genetic modifier that rescues Aβ42-mediated neuronal cell death. N-acetylation is a post translational modification of proteins, and it takes place at the beginning of translation [47, 49]. *Drosophila* gene *CG11539* encodes for a NAT family protein, Mnat9, which is related to human NAT9 [49]. Mnat9 acetylates the N-terminus of alpha and beta Tubulin, subunits of microtubules. Mnat9 is ubiquitously expressed at a basal level in the larval eye imaginal disc. RNA seq data from the *Drosophila* model organism Encyclopedia of DNA elements (modENCODE) indicates that *Mnat9* is also expressed at a basal level in different stages of development [77]. Moreover, its expression is low/ basal level in all adult cell types as shown by Fly Atlas scRNA-seq [78]. This is the first study that reports the role of Mnat9 in modifying cell death observed in our *Drosophila* AD model. The phenotypic data strongly suggest that Mnat9 plays a role in downregulating neuronal cell death in AD-like neuropathology. Additionally, in AD, neurons die, and their guidance and targeting are impaired, which is an underlying cause of synaptic failure [71, 79, 80]. Axonal targeting defects, and neuronal dysfunction have been implicated in *Drosophila* AD model [8, 71, 81, 82]. We identified that Mnat9 can restore the axonal targeting of retinal neurons as evident from chaoptin staining. It has been reported that *Mnat9* is necessary for the stabilization of microtubules [49]. Hence, *Mnat9* rescues both phenotypic and the axonal targeting defects observed in AD. It is known that axonal misguidance or defective targeting in tissues results in neuronal cell death [80]. A similar mechanism may be involved in Aβ42 (*GMR* > *Aβ42*) accumulation, and has been reported earlier to trigger a severe neuronal cell death response [33, 71]. Based on our studies, Mnat9 may reduce this cell death and ROS production observed in *GMR* > *Aβ42* model of AD in flies. Therefore, Mnat9 may be providing survival cues to the neurons that are expressing high levels of human Aβ42 polypeptides.

Currently, Mnat9 is also known for its role in protecting cells from undergoing cell death by inhibiting the JNK signaling pathway [49]. Mnat9 catalyzes N-terminal acetylation of alpha- and beta- Tubulins of microtubules in vitro through its N-acetyltransferase domain. Interestingly, we found that N-terminal acetylation activity of Mnat9 in *Drosophila* might not be required for its neuroprotective function in Aβ42 plaques. We confirmed this in flies where the Acetyl-CoA binding site of both *Drosophila* Mnat9 and human hNAT9 was mutated. Surprisingly, the overexpression of mutated *Mnat9* as well as *hNAT9* exhibited a significant rescue in *GMR* > *Aβ42* flies instead of enhancing the reduced eye phenotype. This finding suggests that acetylation defective Mnat9 is equivalently functional as wild-type Mnat9 in terms of its neuroprotective function in our AD model. Moreover, acetylated Tubulin levels were not significantly modulated when *Mnat9* was overexpressed in *GMR* > *Aβ42* background. Our data suggests that N-terminal acetylation activity of Mnat9 is not required for preventing neurodegenerative phenotype of *GMR* > *Aβ42* flies.

Mnat9 belongs to the NAT family that stabilizes the microtubules. N-acetyltransferases are evolutionarily conserved. *Drosophila Mnat9* and human *hNAT9* exhibit approximately 54% DNA sequence identity and 49% protein identity respectively [49] and is expressed both in human brain and gonads [83]. Moreover, overexpression of human *hNAT9* causes significant rescue in *GMR* > *Aβ42* flies suggesting that *Mnat9* and human *hNAT9* are functionally conserved as well. Previously, it has been shown there is functional conservation of human NAT9 and Mnat9 in wing development [49]. Currently, the role of *hNAT9* in humans is not clear. It was proposed that defective regulation of NAT9 may serve as a susceptibility factor for psoriasis (OMIM 177900), a chronic inflammatory skin disorder [84]. Furthermore, chronic inflammation has been increasingly linked to age related neurodegenerative disorders as well as other diseases like diabetes [85]. Our studies show that hNAT9 rescues *GMR* > *Aβ42* phenotype. Thus, there is a strong possibility that this interaction might extend to higher organisms as well.

Mnat9 is the NAT family protein that regulates JNK signaling during development. Furthermore, knockdown of *Mnat9* results in the aberrant activation of JNK signaling pathway [49]. In AD, accumulation of amyloid plaques leads to aberrant activation of the JNK signaling pathway resulting in cell death [8, 29, 30]. Therefore, we analyzed pJNK levels in eye imaginal discs when *Mnat9* was overexpressed in *GMR* > *Aβ42* flies and found significant decrease in pJNK levels. Our results also support that Mnat9 regulates JNK signaling pathway. Hence, our results indicate that gain-of-function of *Mnat9* ameliorates the neurodegenerative phenotype of *GMR* > *Aβ42* fly eye by downregulating JNK pathway.

It would be interesting in the future to investigate the functional motifs that mediate JNK- Mnat9 interactions that likely underlie the genetic modifications observed in our AD studies. Our study explores the Mnat9-JNK interaction in AD, which presents an interesting opportunity of targeting AD progression by evaluating inhibitors of cell death signals (like JNK) downstream of amyloid plaques/ neurofibrillary tangles. This Mnat9-JNK interaction can modify cell death observed in AD. It would be interesting to explore if blocking JNK pathway by JNK inhibitor(s) can rescue the neurodegenerative phenotype caused by knockdown of *Mnat9* in the background of *GMR* > *Aβ42*. One of the physical interactors of hNAT9 is the mitogen-activated protein kinase 6 (MAPK6) identified by yeast two-hybrid system [86]. Moreover, MAPK6 is an interacting partner of c-Jun and regulates Activator Protein-1 (AP1) activity [87]. Since JNK signaling is involved in many functions during development, the chemical inhibition of this pathway may not be a useful strategy. Furthermore, misregulation of NAT9 may serve as a susceptibility factor for psoriasis and inflammation. hNATs have also been implicated in cancer but the role of NAT9 in cancer remains unknown. Based on the effects of genetic interactions between *hNAT9* and Hippo signaling in the fly [50], it would be interesting to explore whether upregulation of hNAT9 is associated with high grade tumors [49]. Interestingly, we have shown that a positive feedback loop between Hippo signaling and JNK signaling pathway regulates Aβ42 mediated neurodegeneration [21, 29]. Thus, there is an interesting possibility that hNAT9 may be an attractive therapeutic target both in the context of cancer as well as neurodegenerative disorders. Our data suggest that *Mnat9* and *hNAT9* are functionally conserved. Interestingly, remodelin, a putative small molecule inhibitor of N-acetyl transferase 10 (NAT10), is a promising inhibitor which has shown preclinical efficiency in models of premature aging disease Hutchionson-Gilfords Progeria Syndrome (HGPS) [88]. Therefore, further exploration of human NAT9 (*hNAT9*) in mammalian model systems can help shed light on the etiology of AD as well as the potential role of hNAT9 as a druggable target of AD.

## Supplementary information

Supplementary material

Original data

## Data Availability

All data generated or analyzed during this study are included in this published article [and its supplementary information files].
