# Supplementary material for: N-Acetyltransferase 9 ameliorates Aβ42-mediated neurodegeneration in the Drosophila eye
Source: Cell Death Dis. 2023 Jul 28;14(7):478. doi: 10.1038/s41419-023-05973-z (PMC10382493; doi:10.1038/s41419-023-05973-z)
Supplement: Supplementary file 1 — Supplementary material [file 41419_2023_5973_MOESM1_ESM.docx]

**Supplementary Data**


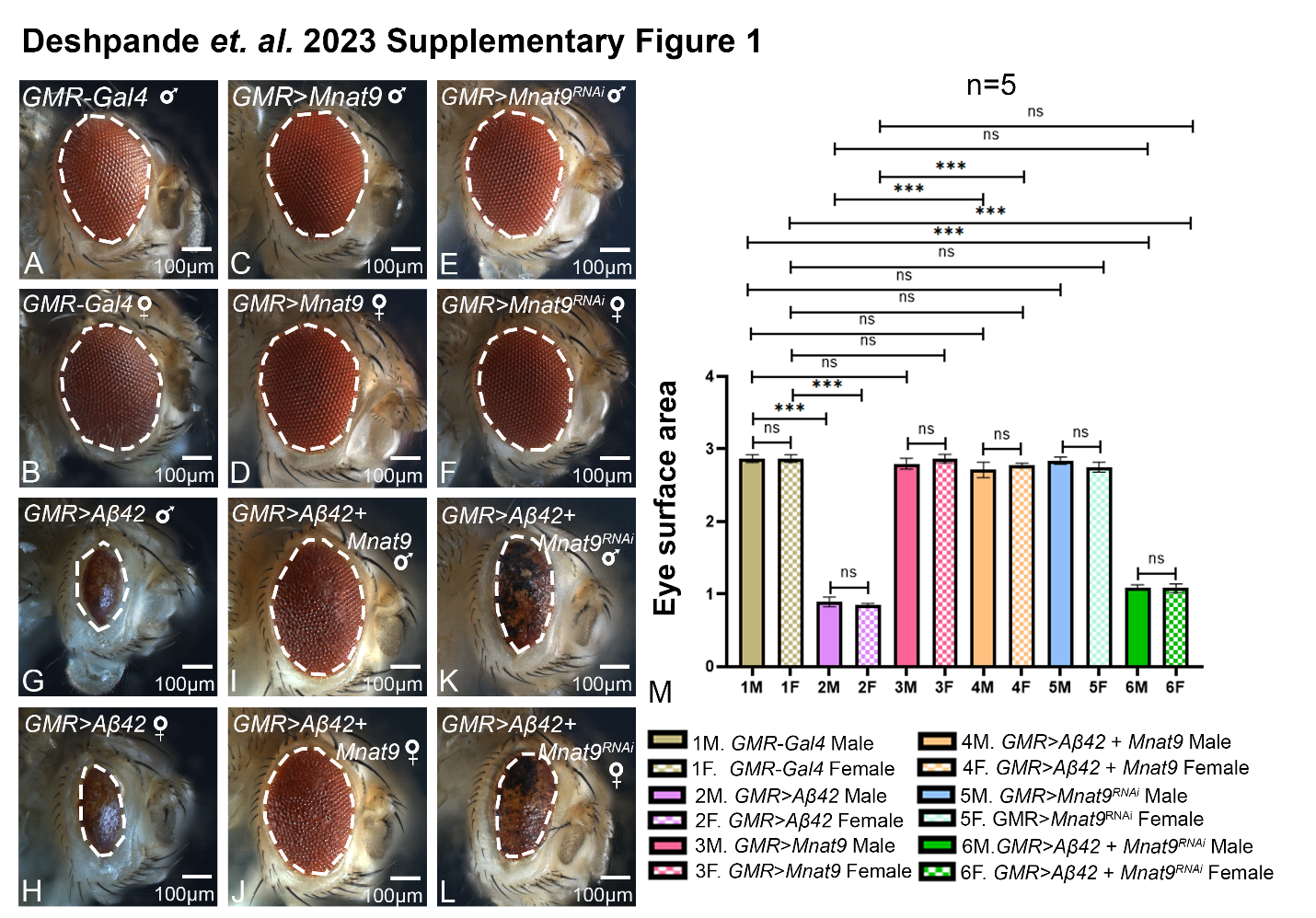


**Supplementary Figure 1:** **Mnat9 doesn’t exhibit any sex- differences in rescuing Aβ42-mediated neurodegeneration.** Adult eye of (A) *GMR-Gal4* male, (B) *GMR-Gal4* female (C) *GMR>Mnat9* male (D) *GMR>Mnat9* female (E) *GMR>Mnat9^RNAi^* male (F) *GMR>Mnat9^RNAi^* female that serve as a control and do not show any sex specific difference. (G) *GMR>Aβ42* male and (H) *GMR>Aβ42* female results in highly reduced glazed adult eye phenotype. (I) *GMR>Aβ42+Mnat9* male and (J) *GMR>Aβ42*+*Mnat9* female results in significant rescue whereas (K) *GMR>Aβ42+Mnat9^RNAi^* male and (L) *GMR>Aβ42+Mnat9^RNAi^* male enhances *GMR>Aβ42* neurodegenerative phenotype. (M) Quantitative analyses of area of the eye. The surface area of the eye (within white dotted line) was calculated using Image J. The surface area of eyes of five flies were calculated per genotype (1. *Canton-S,* 2. *GMR-Gal4,* 3. *GMR>Aβ42,* 4. *GMR>Mnat9,* 5. *GMR>Aβ42+Mnat9,* 6. *GMR> Mnat9^RNAi^ and* 7. *GMR>Aβ42+Mnat9^RNAi^*). Statistical analysis was performed using the student’s t-test for independent samples. Error bars show standard error of mean (mean ± SEM), and symbols above the error bar signify as *** p-value <0.001, ** p-value <0.01, * p-value <0.05, and not significant (n. s.) p-value >0.05 respectively. Scale bar= 100 μm.


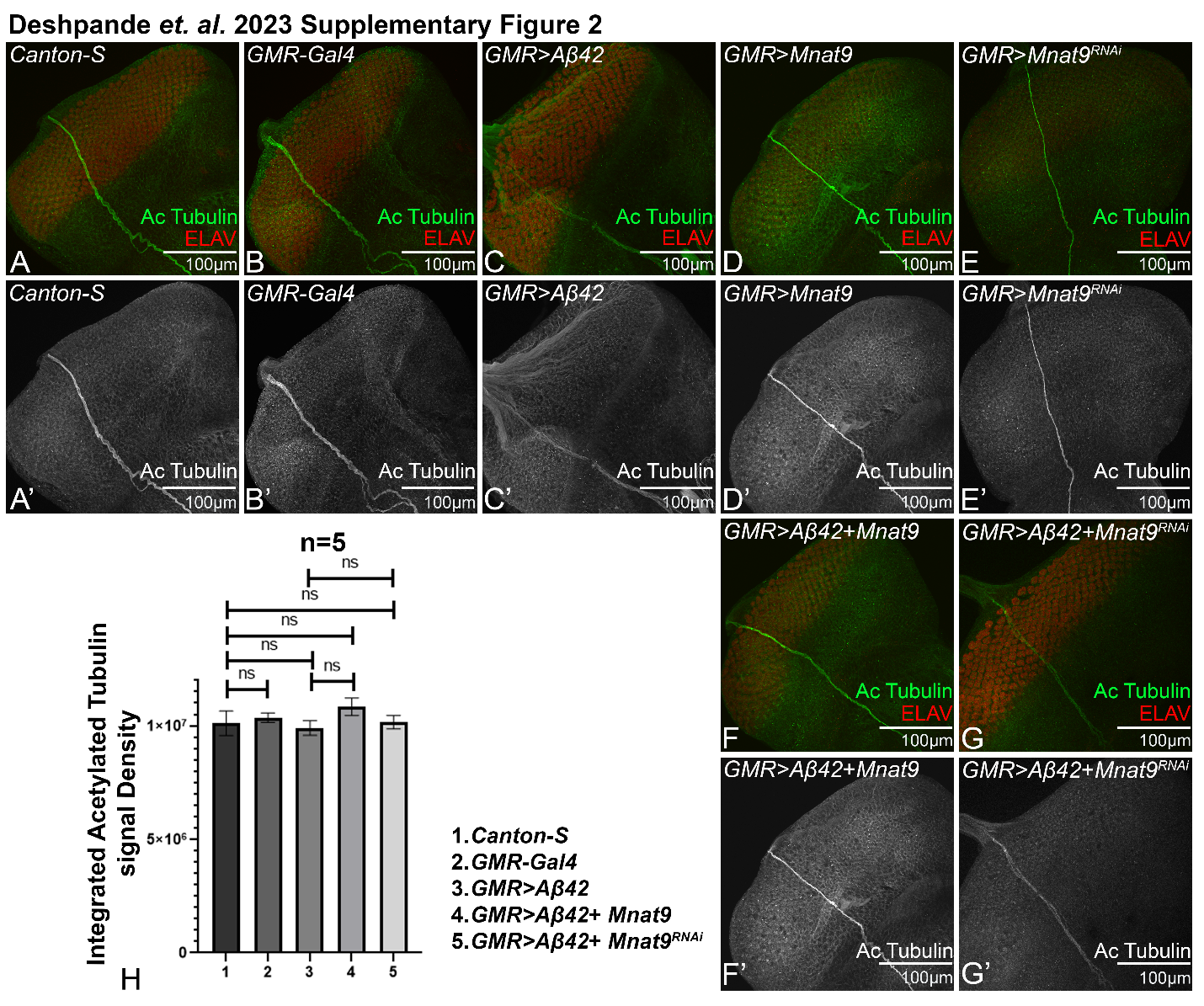


**Supplementary Figure 2: *Mnat9* does not affect the acetylated Tubulin levels.** The third instar larval eye imaginal discs were stained with the pro-neural marker embryonic lethal abnormal vision (ELAV; shown in red) and an anti-Acetylated Tubulin (Ac-Tubulin) antibody (green or gray). (A-G) shows confocal images of eye discs for all markers (Ac Tubulin, ELAV), whereas the Ac Tubulin expression alone (gray) is shown in panels (A’-G’). (A-G, A’-G’) Eye discs of all genotypes were compared and do not show significant change in the acetylated Tubulin levels except for slight increased expression of acetylated Tubulin in *GMR>Aβ42*+*Mnat9* (n=5; p=0.02). (H) Bar graph shows the intensity of acetylated Tubulin levels. The acetylated Tubulin levels were statistically quantified within the region of interest by Fiji/ ImageJ software. Number of samples=5 was used per genotype for the calculation (1. *Canton-S,* 2*. GMR-Gal4,* 3*. GMR>Aβ42,* 4. *GMR>Aβ42+Mnat9,* 5. *GMR>Aβ42+Mnat9^RNAi^*). Statistical analysis was performed using the student’s t-test for independent samples. Error bars show standard error of mean (mean ± SEM), and symbols above the error bar signify as *** p-value <0.001, ** p-value <0.01, * p-value <0.05, and not significant (n. s.) p-value >0.05 respectively. The orientation of all imaginal discs is identical with posterior to the left and dorsal up. Scale bar= 100 μm.


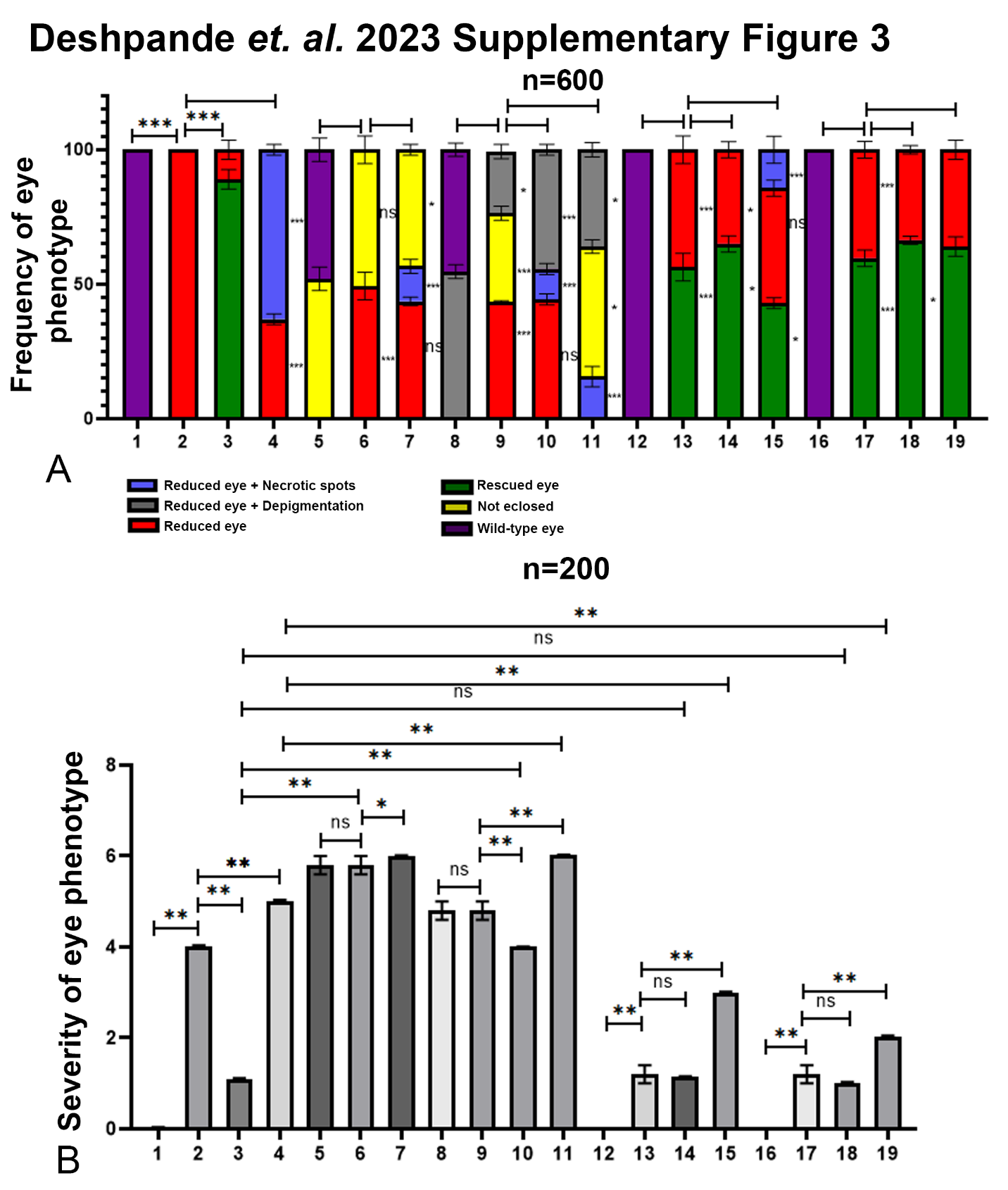
**Supplementary Figure 3: Frequency and severity of eye phenotype representing Mnat9 regulates JNK signaling pathway in developing eye.** (A) Bar graph shows frequency of eye phenotype(s). Six hundred flies were counted for calculating the frequency for each genotype (1. *GMR-Gal4*, 2. *GMR>Aβ42,* 3*. GMR>Aβ42+ Mnat9*, 4*. GMR>Aβ42+ Mnat9^RNAi^,* 5. *GMR>hep^Act^* 6. *GMR>Aβ42+hep^Act^,* 7*. GMR>Aβ42+ Mnat9+hep^Act^,* 8. *GMR>jun^aspV7^,* 9. *GMR>Aβ42+ jun^aspV7^,* 10*. GMR>Aβ42+ Mnat9+ jun^aspV7^,* 11. *GMR>Aβ42+ Mnat9^RNAi^+ jun^aspV7^,* 12*. GMR>bsk^DN^,* 13. *GMR>Aβ42+bsk^DN^,* 14. *GMR>Aβ42+ Mnat9+ bsk^DN^,* 15. *GMR>Aβ42+ Mnat9^RNAi^+ bsk^DN^,* 16*. GMR>puc,* 17. *GMR>Aβ42+puc,* 18. *GMR>Aβ42+ Mnat9+ puc,* 19. *GMR>Aβ42+ Mnat9^RNAi^+puc*). Statistical analysis was performed using the student’s t-test for independent samples. (B) Quantitative analyses of severity score of eye degenerative phenotype(s). Flies from each genotype were randomly selected for scoring according to criteria described in the methods section. Comparisons were made using non-Parametric: Mann Whitney t-Test. Error bars in all graphs show standard error of mean (mean ± SEM), and symbols above the error bar signify as *** p-value <0.001, ** p-value <0.01, * p-value <0.05, and not significant (n. s.) p-value >0.05 respectively.


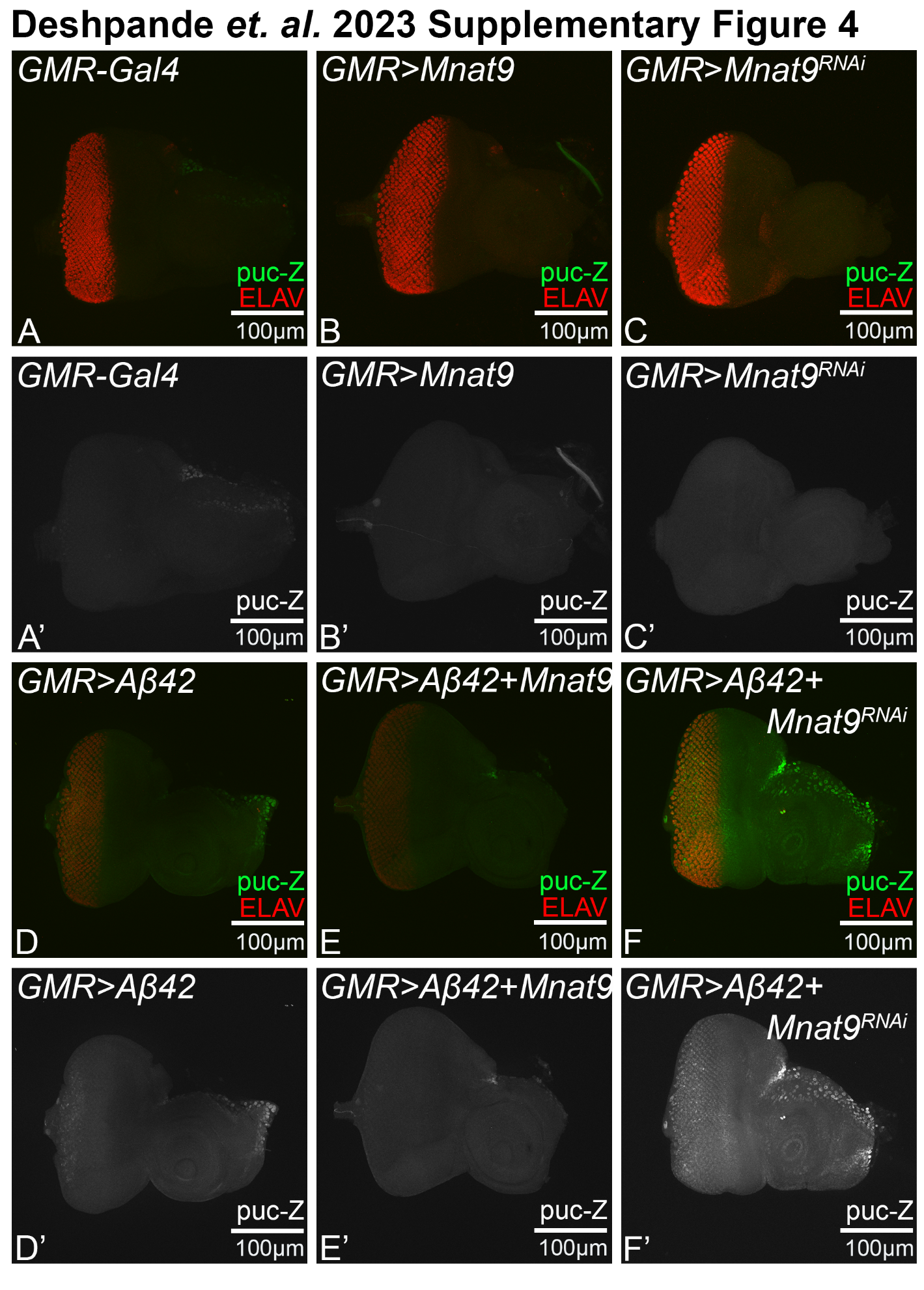
**Supplementary Figure 4:**  **Misexpression of *Mnat9* modulates the *puc* LacZ expression in the eye disc.** The third instar larval eye imaginal discs exhibit the expression of *puc* using lacZ reporter (red) (A, A’) *GMR-Gal4* (B, B’) *GMR*>*Mnat9* and (C, C’) *GMR>Mnat9^RNAi^*, serve as controls, with no expression of *puc-*LacZ. (D, D’) The expression of *puc-*LacZ increases dramatically in *GMR>Aβ42* eye discs. (E, E’) Whereas the *puc-*LacZ expression is downregulated in *GMR>Aβ42*+*Mnat9.* (F, F’) The downregulation of *Mnat9* significantly increases the *puc* LacZ expression. The orientation of all imaginal discs is identical with posterior to the left and dorsal up. Scale bar= 100 μm.


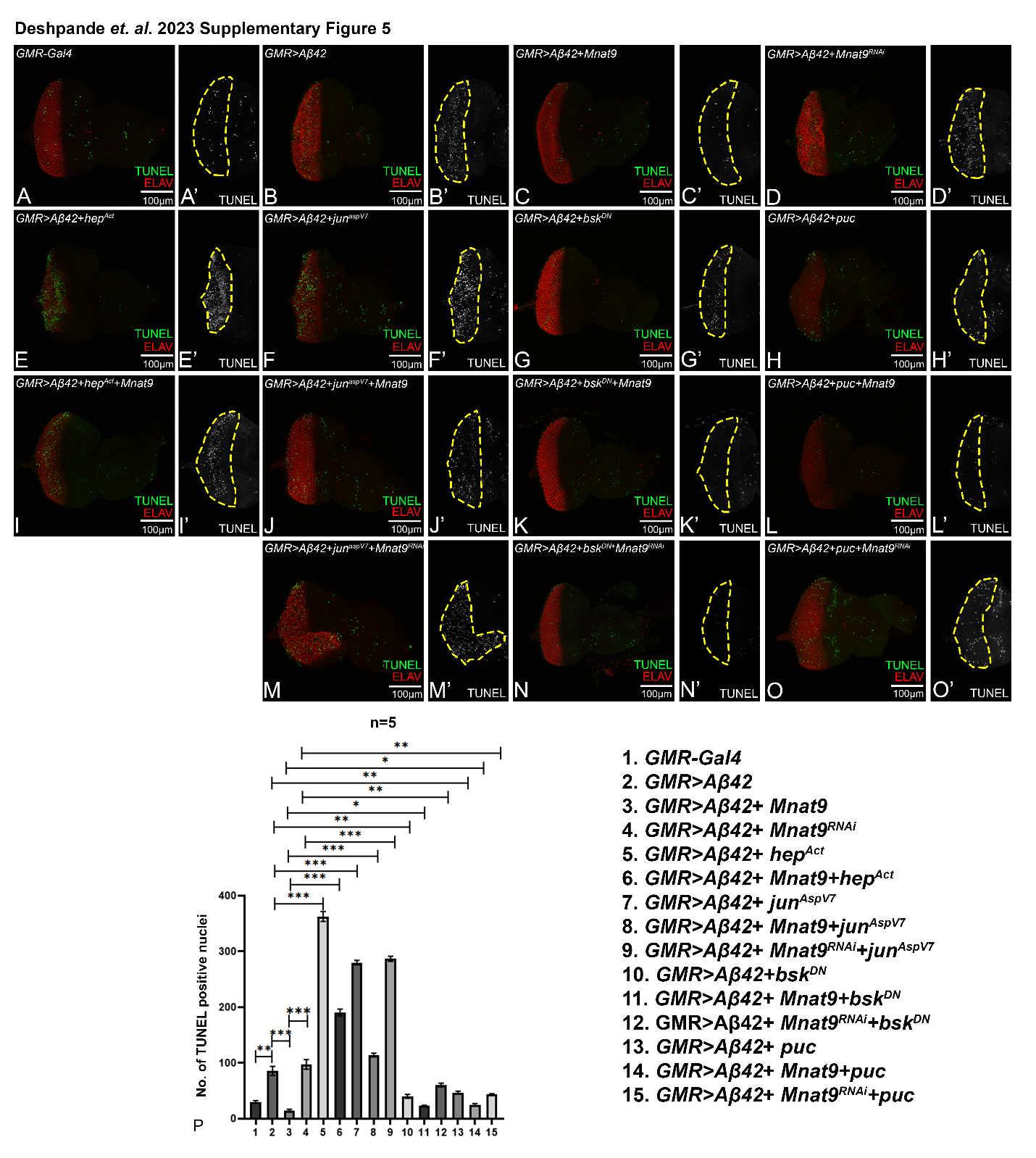
**Supplementary Figure 5: Gain-of-function of *Mnat9* rescues Aβ42-mediated neuronal cell death by downregulating JNK signaling in the fly eye.** (A-O) Eye discs stained for proneural marker ELAV (red) marks the nuclei of retinal neurons and TUNEL (green) to mark the dying nuclei. (A’-O’) Split channel shows TUNEL expression in gray scale mode. (A, A’) *GMR-Gal4* eye imaginal discs show a fewer TUNEL positive nuclei. (B, B’) *GMR>Aβ42* eye discs shows significant increase in TUNEL positive nuclei. (C, C’) *GMR>Aβ42*+*Mnat9* shows significant decrease in the cell death as compared to *GMR>Aβ42*. (D, D’) *GMR>Aβ42*+*Mnat9^RNAi^* shows elevated levels of TUNEL positive nuclei. (E, E’) *GMR>Aβ42+hep^Act^* and (F, F’) *GMR>Aβ42+jun^aspV7^* result in a dramatic increase in TUNEL positive nuclei. (I, I’) *GMR>Aβ42+Mnat9+hep^Act^* and (J, J’) *GMR>Aβ42+Mnat9+jun^aspv7^* result in decreased TUNEL positive nuclei. However, (G, G’) *GMR>Aβ42+bsk^DN^* and (H, H’) *GMR>Aβ42+puc* result in significant decrease in TUNEL positive nuclei as compared to *GMR>Aβ42*. (K, K’) *GMR>Aβ42+Mnat9+bsk^DN^* and (L, L’) *GMR>Aβ42+Mnat9+puc* showed significant decrease in TUNEL positive nuclei. (M, M’) *GMR>Aβ42+Mnat9^RNAi^+jun^aspv7^* results in significantly increased cell death. (N, N’) *GMR>Aβ42+Mnat9^RNAi^+bsk^DN^* and (O, O’) *GMR>Aβ42+Mnat9^RNAi^+puc* showed significant decrease in TUNEL positive nuclei as compared to *GMR>Aβ42+Mnat9^RNAi^*. (A’-O’, P) Bar graph represents dying nuclei. The TUNEL positive nuclei were statistically quantified within the red dotted line- the region of interest. Number of samples=5 was used per genotype for the calculation (1. *GMR-Gal4,* 2*. GMR>Aβ42,* 3. *GMR>Aβ42+Mnat9,* 4. *GMR>Aβ42+Mnat9^RNAi^,* 5. *GMR>Aβ42+hep^Act^,* 6. *GMR>Aβ42+Mnat9+hep^Act^,* 7. *GMR>Aβ42+jun^aspV7^*, 8. *GMR>Aβ42+Mnat9+jun^aspV7^,* 9. *GMR>Aβ42+ Mnat9^RNAi^+jun^aspV7^,* 10*. GMR>Aβ42+bsk^DN^,* 11. *GMR>Aβ42+Mnat9+ bsk^DN^,* 12*. GMR>Aβ42+ Mnat9^RNAi^+ bsk^DN^,* 13. *GMR>Aβ42+ puc,* 14. *GMR>Aβ42+ Mnat9+ puc,* 15. *GMR>Aβ42+ Mnat9^RNAi^+ puc*). Statistical analysis was performed using the student’s t-test for independent samples. Error bars show standard mean of error (mean ± SEM), and symbols above the error bar signify as *** p-value <0.001, ** p-value <0.01, * p-value <0.05, and not significant (n. s.) p-value >0.05 respectively. The orientation of all imaginal discs is identical with posterior to the left and dorsal up. Scale bar= 100 μm.
