# Supplementary material for: N-Acetyltransferase 9 ameliorates Aβ42-mediated neurodegeneration in the Drosophila eye
Source: Cell Death Dis. 2023 Jul 28;14(7):478. doi: 10.1038/s41419-023-05973-z (PMC10382493; doi:10.1038/s41419-023-05973-z)
Supplement: Supplementary file 2 — Original data [file 41419_2023_5973_MOESM2_ESM.docx]

Uncropped Western blots

1. Blot 1_Tubulin (Molecular weight=55kDa)

1 2 3 4 5 6 7

50kDa

Lane 1: Bio-Rad Precision Plus

Protein Kaleidoscope standard

Lane 2: *GMR-Gal4*

Lane 3: *GMR>Aβ42*

Lane 4: *GMR>Mnat9*

Lane 5: *GMR>Aβ42+ Mnat9*

Lane 6: *GMR>Mnat9^RNAi^*

Lane 7: *GMR>Aβ42+ Mnat9^RNAi^*

37kDa


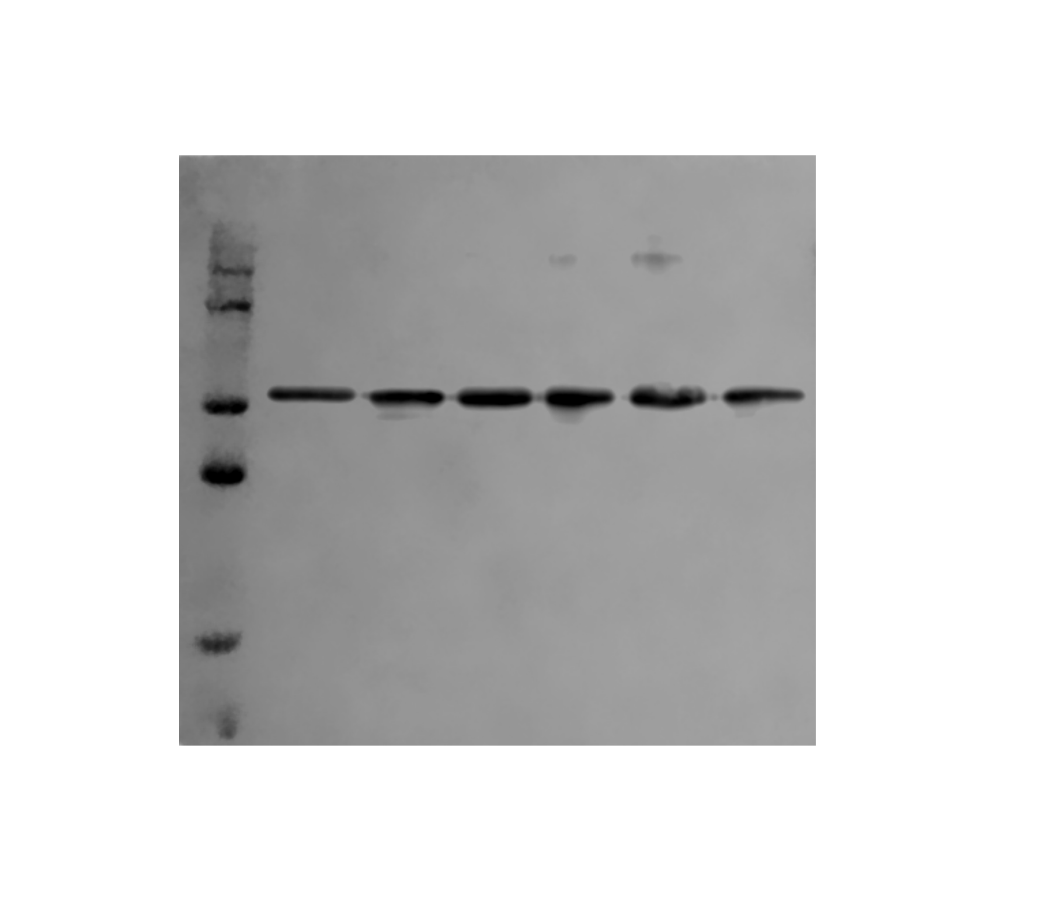


Tubulin

1. Blot 1 pJNK (Molecular weight=46kDa)

1 2 3 4 5 6 7


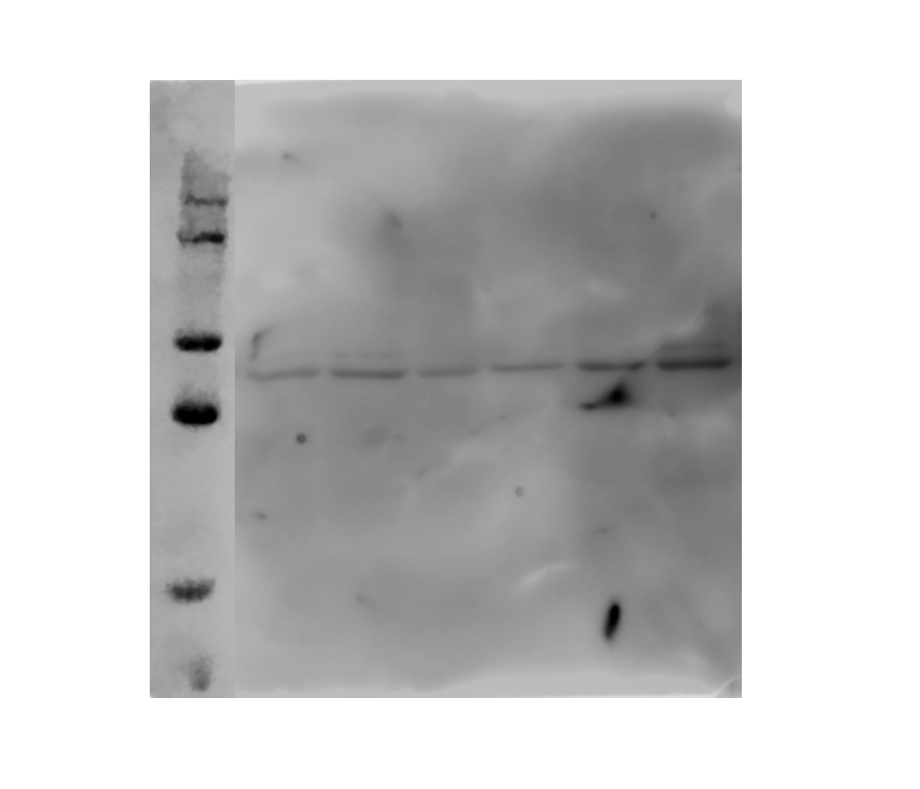


37kDa

50kDa

Lane 1: Bio-Rad Precision Plus

Protein Kaleidoscope standard

Lane 2: *GMR-Gal4*

Lane 3: *GMR>Aβ42*

Lane 4: *GMR>Mnat9*

Lane 5: *GMR>Aβ42+ Mnat9*

Lane 6: *GMR>Mnat9^RNAi^*

Lane 7: *GMR>Aβ42+ Mnat9^RNAi^*

pJNK

1. Blot 2_Tubulin (Molecular weight=55kDa), pJNK (Molecular weight=46kDa)

Tubulin

pJNK

1 2 3 4 5 6 7

37kDa

50kDa

Lane 1: Bio-Rad Precision Plus

Protein Kaleidoscope standard

Lane 2: *GMR-Gal4*

Lane 3: *GMR>Aβ42*

Lane 4: *GMR>Mnat9*

Lane 5: *GMR>Aβ42+ Mnat9*

Lane 6: *GMR>Mnat9^RNAi^*

Lane 7: *GMR>Aβ42+ Mnat9^RNAi^*


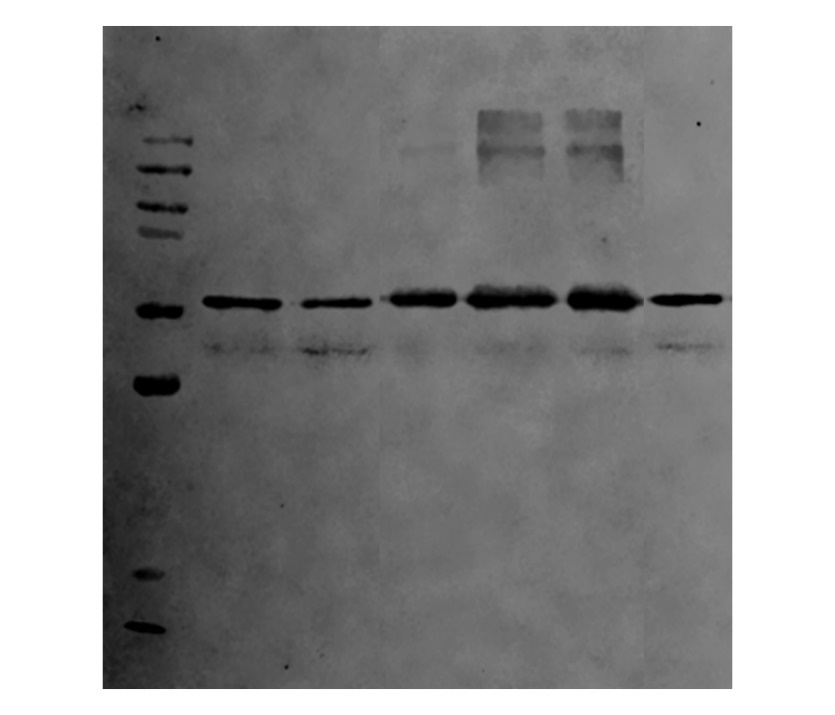


1. Blot 3_Tubulin (Molecular weight=55kDa), pJNK (Molecular weight=46kDa)

Tubulin

pJNK

1 2 3 4 5 6 7

Lane 1: *GMR-Gal4*

Lane 2: *GMR>Aβ42*

Lane 3: *GMR>Mnat9*

Lane 4: *GMR>Aβ42+ Mnat9*

Lane 5: *GMR>Mnat9^RNAi^*

Lane 6: *GMR>Aβ42+ Mnat9^RNAi^*

Lane 7: Bio-Rad Precision Plus Protein Kaleidoscope standard


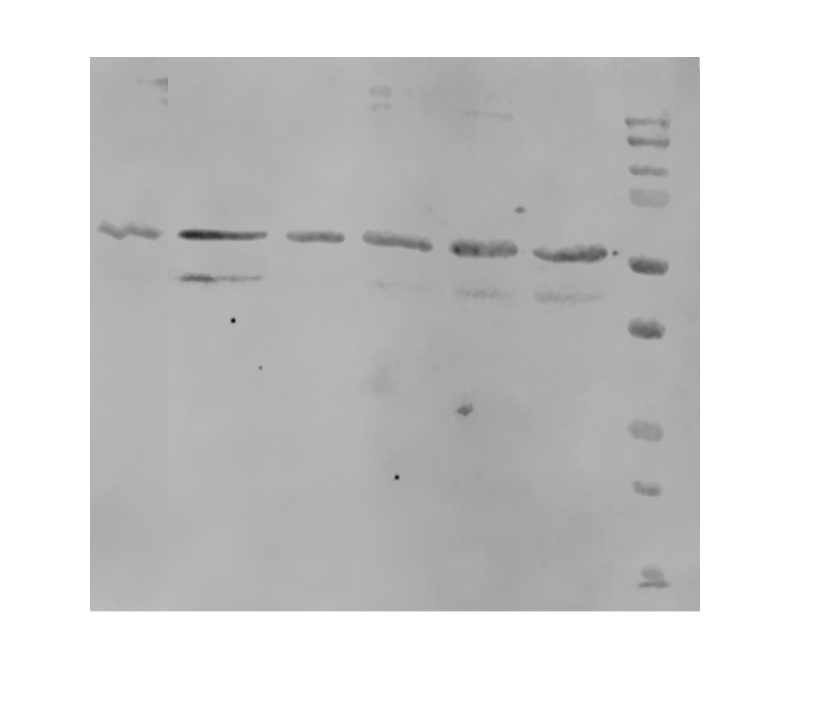


50kDa

37kDa
